# Adenovirus-Inspired Virus-like-Particles Displaying Melanoma Tumor Antigen Specifically Target Human DC Subsets and Trigger Antigen-Specific Immune Responses

**DOI:** 10.3390/biomedicines10112881

**Published:** 2022-11-10

**Authors:** Solène Besson, David Laurin, Cyrielle Chauvière, Michel Thépaut, Jean-Philippe Kleman, Mylène Pezet, Olivier Manches, Franck Fieschi, Caroline Aspord, Pascal Fender

**Affiliations:** 1 Institut de Biologie Structurale, CEA, CNRS, University Grenoble Alpes, UMR5075, 38042 Grenoble, France; 2 Immunobiology and Immunotherapy in Chronic Diseases, Institute for Advanced Biosciences, Inserm U 1209, CNRS UMR 5309, University Grenoble Alpes, 38000 Grenoble, France; 3 R&D Laboratory, Etablissement Français du Sang Auvergne-Rhône-Alpes, 38000 Grenoble, France

**Keywords:** adenovirus, vaccine platform, immunotherapy, melanoma, C-type lectin receptors

## Abstract

Virus-like particles constitute versatile vectors that can be used as vaccine platforms in many fields from infectiology and more recently to oncology. We previously designed non-infectious adenovirus-inspired 60-mer dodecahedric virus-like particles named ADDomers displaying on their surface either a short epitope or a large tumor/viral antigen. In this work, we explored for the first time the immunogenicity of ADDomers exhibiting melanoma-derived tumor antigen/epitope and their impact on the features of human dendritic cell (DC) subsets. We first demonstrated that ADDomers displaying tumor epitope/antigen elicit a strong immune-stimulating potential of human DC subsets (cDC2s, cDC1s, pDCs), which were able to internalize and cross-present tumor antigen, and subsequently cross-prime antigen-specific T-cell responses. To further limit off-target effects and enhance DC targeting, we engineered specific motifs to de-target epithelial cells and improve DCs’ addressing. The improved engineered platform making it possible to display large antigen represents a tool to overcome the barrier of immune allele restriction, broadening the immune response, and paving the way to its potential utilization in humans as an off-the-shelf vaccine.

## 1. Introduction

Recent years have seen the development of a variety of immunotherapies in oncology with various success depending on the cancer type and state [1,2,3]. In 2013, Chen DS and Mellman I [4] presented a cancer-immunity cycle explaining the fundamental steps by which the immune system manages to kill cancer cells. This cycle highlights the fundamental role of dendritic cells in the triggering of the anti-tumoral immune response. The aim of any immunotherapies is to drive this cycle and increase one of those steps.

For instance, the blocking of the immune checkpoint PD-1 occurs at the last step of this cycle when cytotoxic T cells destroy cancer cells. On the other hand, cancer vaccines can have an action at the very beginning of this cycle by activating the DCs, leading to the differentiation of T cells into tumor-specific cytotoxic T lymphocytes (CTL), triggering the death of cancerous cells [5]. Indeed, a cancer vaccine’s goal is to stimulate the patient’s immune system against the vaccine’s carried epitopes or antigens. Vaccines are captured by DCs and epitopes/antigens are internalized and presented (cross-presentation) through peptide/HLA complexes to naive T cells triggering their activation and differentiation into antigen-specific CD4+ or CD8+ T cells in lymph nodes (cross priming). Both subsets have different roles: CD4+ T cells are required for CD8+ T cells activation, which will then exhibit their cytotoxic activity. Once activated, CD8+ T cells will travel from the lymph node to the tumor site through the blood circulation. At the tumor site, activated CD8+ T infiltrate the tumor and recognize the cancer cells thanks to the antigens expressed at their surface through peptide/HLA complexes. Then, they can kill the cancer cells thanks to their cytotoxic activity [4]. Many cancer vaccines are currently under evaluation at preclinical or clinical stages using different strategies, from tumor cell vaccines to genetic vaccines [6,7]. Among genetic vaccines there are viral vectors, such as adenovirus. They can be used in two different manners: either as oncolytic viruses, meaning that they would replicate only inside tumor cells, triggering their death, or as a component of a vaccine to stimulate both innate and adaptive immunity, thus triggering an anti-tumoral response [8]. Adenoviruses are widely used in the clinic; more than 100 clinical trials in Europe and almost 300 in the USA are ongoing. Most of them use the full adenovirus as the vector either encoding a tumor antigen, a cytokine or just engineered as oncolytic viruses [9,10,11]. We have previously reported that a non-infectious virus-like particle (VLP) derived from the human Adenovirus of type 3 and consisting of 60 identical penton base monomers could be exploited to display epitopes of interest on its surface [12,13,14]. In this vaccine platform named ADDomer, exposed loops of the penton base protein were engineered to allow insertion of small peptides such as a linear neutralizing epitope from Chikungunya virus [12]. However, this design did not permit the insertion of structurally complex antigens. To overcome this limitation while keeping the immunogenic advantage of ADDomer, we redesigned this platform by genetically inserting a SpyTag. Thanks to that, spontaneous display of large and structurally complex antigens with potential post-translational modifications such as the SARS-CoV-2 Receptor Binding Domain (RBD) or a model antigen ovalbumin fused to the Spycatcher was achieved. In infectiology, this system displaying the RBD has proven to be highly effective to elicit potent neutralizing antibodies against SARS-CoV-2 in mice [15]. In oncology, ADDomers exhibiting the ovalbumin antigen have also proven to be of great efficiency in controlling melanoma B16-OVA in mice [16].

In this study, we explored in vitro the interactions of a cancer vaccine platform ADDomer displaying human melanoma tumor antigen (MelanA) and epitope (A2L) with human DC subsets (cDC2s, cDC1s, pDCs). We deciphered the ability of DCs to internalize such ADDomers, to cross-present associated epitopes/antigens, and to subsequently trigger antigen-specific T-cell responses. To further limit off-target effects and enhance DC targeting, we engineered specific motifs to de-target un-targeted cells (epithelial cells) and improve DCs’ addressing.

## 2. Materials and Methods

### 2.1. Baculovirus Production

The baculovirus expression system was used for the production of different ADDomers (ADD A2L, ADD ST, ADD KGE, and ADD RGD), as well as for the Spike Receptor Binding Domain of SARS-CoV-2 fused to the SpyCatcher (RBD-SC) and the MelanA tumor antigen fused to the SpyCatcher (MelA-SC). Synthetic DNA (Genscript, Rijswijk, The Netherlands) was cloned in the pACEBac1 using the restriction sites BamH I and Hind III. For RBD-SC, the RBD sequence (320–554) was cloned upstream of the SC, and a 6His-Tag was added in the C-terminus of SC. The same was performed for the MelA-SC. The RBD-SC was secreted using the melittin signal peptide present in the vector. Recombinant baculoviruses were made by transposition with an in-house bacmid expressing yellow fluorescent protein, as previously described [12]. Baculoviruses were amplified on Sf21 cells at low multiplicity of infection (MOI) and after two amplification cycles were used to infect insect cells for 64 to 72 h at high MOI. For all proteins except for RBD-SC, the infected cells were pelleted and recovered. For RBD-SC, cells were discarded, and the supernatant was saved.

### 2.2. Protein Purification

#### 2.2.1. ADDomers’ Purification

The ADDomers were purified according to classical protocol [15]. Briefly, after lysis of the insect cell pellet by three cycles of freeze-thaw in the presence of complete protease inhibitor cocktail (Roche, Paris, France) and removal of debris, the lysate was loaded onto a 20% to 40% sucrose density gradient. The gradient was centrifuged for 18 h at 4 °C on an SW 41 Ti rotor in a Beckman XPN-80 ultracentrifuge. The dense collected fractions at the bottom of the tubes were dialyzed against HEPES 10 mM pH 7.4, NaCl 150 mM, and then loaded onto a Macroprep Q cartridge (Bio-Rad, Paris, France). After elution by a 150 to 600 mM linear NaCl gradient in HEPES 10 mM pH 7.4, ADDomer-containing fractions were checked by SDS-PAGE and concentrated on Amicon (MWCO: 100 kDa) with buffer exchange to HEPES 10 mM pH 7.4, NaCl 150 mM.

#### 2.2.2. RBD-SC Purification

The insect cell supernatant was centrifuged after thawing for 15 min at 7500× *g* and loaded onto a HEPES 10 mM pH 7.4 preequilibrated Heparin column (Cytiva, Paris, France) of 5 mL for 500 mL of supernatant. The column was washed with HEPES 10 mM pH 7.4 for 25 mL then eluted for 10 mL with 0 to 500 mM linear NaCl gradient in HEPES 10 mM pH 7.4. The eluate was supplemented with 30 mM imidazole-HCl (pH 7.4) and incubated with Ni-NTA beads (Qiagen, Paris, France), 2 mL of beads for 500 mL culture, for at least 1 h at 4 °C under gentle agitation. The beads were then poured into an empty column and the protein was eluted by two column volumes (CV) of 250 mM imidazole in HEPES 10 mM pH 7.4, NaCl 150 mM. It was then submitted to buffer exchange using an Amicon device (MWCO 30 kDa).

#### 2.2.3. MelA-SC Purification

Insect cell pellet was lysed by three cycles of freeze-thaw in the presence of a complete protease inhibitor cocktail (Roche, Paris, France), and the lysate containing the His-tagged MelA-SC was loaded to a 1 mL His GraviTrap prepacked column (GE Healthcare, Paris, France), pre-equilibrated with 20 CV of 150 mM NaCl, 10 mM HEPES pH 7.4 and 10 mM imidazole. Columns were then washed with 20 CV of the same buffer to remove non-specifically bound material. Proteins were eluted with 10 CV of buffer containing 200 mM imidazole. MelA-SC containing fractions were checked by SDS-PAGE and concentrated on Amicon (MWCO: 30 kDa) with buffer exchange to HEPES 10 mM pH 7.4, NaCl 150 mM.

### 2.3. Complex Formation and Labelling

#### 2.3.1. ADD-ST + MelA/RBD-SC (ADD-MelA, ADD KGE RBD and ADD RGD RBD) Complex Formation for Cross Priming and Cross Presentation Experiments

*Covalent complex formation was obtained by incubation of purified* ADD-ST with purified MelA/RBD protein fused to SC (i.e MelA/RBD-SC). Incubation was performed at 25 °C under agitation on a ThermoMixer (Eppendorf, Paris, France) at 300 rpm. RBD-SC ratio and MelA-SC per ADD-ST was fixed to 1:1 (1 MelA/RBD-SC for 1 monomer) in order to avoid steric hindrance between the MelA/RBD-SC proteins on the ADDomer surface.

Left over RBD-SC/MelA-SC which did not bind to the ADDomers were removed thanks to a 20% to 40% sucrose density gradient. The gradient was centrifuged for 6 h at 60,000 rpm, 4 °C on an SW 60 Ti rotor in a Beckman XPN-80 ultracentrifuge. The dense collected fractions at the bottom of the tubes (complex containing fractions i.e., ADD-MelA/ADD KGE RBD/ADD RGD RBD) were checked by SDS-PAGE and concentrated on Amicon (MWCO: 100 kDa) with buffer exchange to HEPES 10 mM pH 7.4, NaCl 150 mM for sucrose removal. (Supplementary Figure S1).

#### 2.3.2. ADD-ST AF647 + MelA/RBD-SC (ADD-MelA AF647, ADD KGE/RGD +/− RBD AF647) Complex Formation for Internalization Experiments

Covalent complex formation was obtained by incubation of purified ADD-ST with purified MelA/RBD protein fused to SC (MelA/RBD-SC). ADD ST, ADD KGE and ADD RGD were first incubated with the fluorochrome NHS-Alexa Fluor 647 (ThermoFisher, Paris, France) overnight at 25 °C under agitation on a Thermomixer at 300 rpm. The NHS moiety forms a covalent bond with the ADDomers’ lysins terminal NH_2_ moiety. ADDomers AF647 were then incubated with ethanolamine (1 mM final concentration) for 4 h at 25 °C under agitation on a Thermomixer at 300 rpm. Finally, incubation with the MelA/RBD-SC was performed overnight at 25 °C under agitation on a Thermomixer at 300 rpm. RBD-SC ratio and MelA-SC per ADD-ST was fixed to 1:1 (1 MelA/RBD-SC for 1 monomer) in order to avoid steric hindrance between the MelA/RBD-SC proteins on the ADDomer surface. Ethanolamine allows the blockage of left-over NHS-AF647 in order to prevent its fixation to RBD-SC/MelA-SC (Supplementary Figure S2). Left-over RBD-SC/MelA-SC were removed as described above.

### 2.4. Cell Preparation and Culture

#### 2.4.1. Transformed Cell Lines

All experiments were performed in complete RPMI medium (RPMI-1640 Glutamax (Gibco^®^, Paris, France) enriched with 20 μg/mL Gentamicin, 1% non-essential amino-acids both from (Invitrogen™, France) and 1 mM sodium pyruvate (Sigma, Paris, France)) supplemented with 10% decomplemented fetal calf serum (FCS) (Invitrogen™).

#### 2.4.2. Peripheral Blood Mononuclear Cells (PBMCs) Isolation

Peripheral Blood Mononuclear Cells (PBMCs) were isolated from healthy donor blood and purified by the Ficoll-Hypaque density gradient centrifugation (800× *g*, 20 min) technique (Eurobio, Paris, France). The ring of PBMCs formed at the interface between the plasma and the Ficoll is washed in phosphate buffer saline (PBS), centrifuged at 400× *g* for 8 min before de-plaquettation at 200× *g* for 10 min at 4 °C. PBMC were then numerated and stored frozen at −196 °C in liquid nitrogen until use.

### 2.5. Fixation, Internalization of ADDomer

#### 2.5.1. Evaluation of the Fixation of ADDomers AF647 on DC Subsets by Flow Cytometry

PBMC (2.10^6^ in 250 µL) were incubated with 0.15 µM (1×), 0.5 µM (3×) or 1.5 µM (10×) of each ADDomer AF647 in FACS tubes for 1 h at 37 °C, 5% CO_2_ in RPMI supplemented with 10% FCS or in PBS 1× for tubes treated with EDTA (ADD KGE RBD + EDTA and ADD RGD RBD + EDTA). For tubes treated with EDTA, PBMC (2.10^6^ in 250 µL) were first incubated with EDTA in PBS 1× (20 mM final concentration) for 30 min at RT. Cells were then washed, incubated with the ADDomers AF647 in PBS 1×, and washed with PBS 1× supplemented with 2% FCS. Next, cells were resuspended in 100 μL PBS 2% FCS and incubated for 20 min in the dark with a mixture of Abs conjugated to fluorochromes (anti-human CD45, Lin, HLA-DR, CD11c, BDCA1, BDCA2, Clec9a antibodies) and live&dead staining, allowing us to detect the ADDomers AF647 fixed or internalized by the three alive DCs subsets. Then, cells were washed in 2 mL PBS 2% FCS before being fixed in 300 μL FACS lysing solution 1×. Analyses were performed using LSR II and analyzed with DIVA 8.3 software from BD.

#### 2.5.2. Determination of the Internalization of ADDomers AF647 by DC Subsets by Confocal Microscopy

To determine whether the ADDomers are internalized or just fixed through surface adhesion by the DC subsets, confocal microscopy was performed on panDCs pre-incubated with ADDomers AF647. PanDCs were purified from PBMC using the kit PanDC pre-enrichment kit (StemCell, Paris, France). PanDCs (1.2.10^6^ in 600 µL) were incubated with 0.15 µM of each ADDomer-AF647 in FACS tubes for 1 h 30 at 37 °C, 5% CO_2_ in RPMI supplemented with 10% FCS. Next, cells were washed in PBS 1× and deposited on 3 poly-lysin coated slides (400,000 cells in 200 µL per slide, 1 slide for each three DC subset). Slides were then placed 20 min at 37 °C in the dark for panDC adhesion. Upon removing the medium, slides were incubated with 100 µL of an antibody mix for 10 min at 37 °C in the dark, comprising CD11c, HLA-DR, and either BDCA1, BDCA2 or BDCA3 antibodies. Slides were then washed with 200 µL of PBS 1×, then cells were fixed with 200 µL of PFA 4% for 10 min at 37 °C in the dark. Slides were then washed twice with 200 µL of PBS 1× before being mounted with 10 µL anti-fade mounting medium (Invitrogen). The localization of ADDomers was assessed with a laser spinning disk Andromeda-IMIC inverted microscope (Till-Photonics FEI, Paris, France) equipped with an alpha-Plan-Apochromat 63x/1.46 objective (Zeiss, Paris, France). Images were acquired with an iXon U897 EMCCD camera (Andor, Paris, France) controlled with Live Acquisition Software (FEI). Z series optical sections were collected with a step size of 300 nm over 10 microns. Equatorial section was used to represent internalization. Images were denoised with denoise.ai NIS-Element (Nikon, Paris, France) software and then analyzed with FIJI. Intensities of CD11c (Brilliant Violet 421) and HLA-DR (Brillant Violet 510) markers as well as the subset marker stained with FITC (BDCA 1, 2 or 3) on the cell surface were used to discriminate between the three subsets.

### 2.6. DC Subsets’ Activation and Cytokine Production upon Incubation with ADDomers

#### 2.6.1. PBMCs Preparation/Cell Culture Conditions

PBMCs from two healthy donors were thawed in medium RPMI 20% FCS, centrifuged at 400× *g* for 8 min, then resuspended in medium RPMI 10% FCS and plated (2.10^6^ cells in 1 mL). PBMCs were incubated either in control conditions, with TLR-ligand (TLR-L) mix (Poly IC 30 μg/mL, R848 1 μg/mL and CpGA 1 μM), with peptide MelA_26–35_ (10 μM), ADD Ø or ADDomer A2L (1 μM) for 24 h.

#### 2.6.2. Cell Viability and Costimulatory Molecules Expression Analysis by Flow Cytometry

Supernatants (SN) of each condition were taken and stored at −20 °C for further cytokine production analysis by Luminex. Cells were harvested and wells washed twice with 1 mL RPMI 10% FCS, then centrifuged at 400× *g* for 8 min and resuspended in 100 μL PBS 2% FCS. Cells were then incubated for 20 min in the dark with a mixture of Abs conjugated to fluorochromes allowing us to detect costimulatory molecules (CD40, CD80, CD86), as well as the cell viability (Live & dead marker) on DCs subsets depicted by using anti-human CD45, HLA-DR, Lin, CD11c, BDCA1, 2 and 3 antibodies. Cells were then fixed in 300 μL FACS lysing solution 1×, washed in 2 mL PBS 2% FCS and centrifuged at 600× *g* for 5 min. Cells were next resuspended in 300 μL PBS 2% FCS and stored at 4 °C until acquisition. Analyses were performed using LSR II and analyzed with DIVA 8.3 software from BD-Biosciences.

#### 2.6.3. Cytokine Secretion Analysis by Luminex

IL12p70, IFNα, IFNβ, IFNλ1/IL29 and IFNλ2/IL28 cytokine secretions were measured in culture supernatants by LUMINEX technology using MAGPIX^®^200 Instrument with xPONENT^®^ software (Bio-Rad).

### 2.7. Analysis of DCs’ Ability to Perform the Cross-Presentation of ADDomers-Associated Epitopes/Antigens

#### 2.7.1. PanDC Incubation with ADDomers

PanDCs (mixture of cDC2s, cDC1s and pDCs) were purified from PBMC from two HLA-A0201^+^ donors using the kit PanDC pre-enrichment kit (StemCell.) PanDCs (20,000 cells in 100 µL) were incubated with 1 µM of each ADDomer (empty, A2L, MelA, KGE, KGE RBD, RGD, RGD RBD) or the restricted peptide MelA_26–35_ at 1 μM overnight at 37 °C, 5% CO_2_ in RPMI supplemented with 10% FCS in a 96 well plate.

#### 2.7.2. Conditions of Cross-Presentation

In order to explore if the ADDomers were processed by DCs, a cross presentation assay was performed. Pre-amplified anti-MelA_26–35_ T cells were co-cultured with panDCs pre-incubated with ADDomers in a ratio 5:1 (100,000 pre-amplified anti-MelA_26–35_ T cells for 20,000 panDCs) in 96 wells plate for 20 h in incubator at 37 °C, and 5% CO2. Controls were carried out by incubating the anti-MelA_26–35_ T cells with either RPMI 10% FCS or activation cocktail (PMA/iono). SNs were collected and analyzed by Cytometric Bead Array (CBA provided by BD) to measure the concentration of IFNγ.

#### 2.7.3. Measurement of Cytokines Production by CBA

To determine the amount of IFNγ produced by cells which reflect their ability to recognize the complex HLA/peptide on the antigen presenting cells such as dendritic cells, SNs of the cross-presentation assay were analyzed by CBA. Standard range was prepared from 2500 pg/mL to 0 pg/mL of recombinant IFNγ by cascade dilution 2 by 2. Fluorescent beads (labeled with AlloPhycoCyanin fluorophores APC or APC-Cy7) covered with anti IFNγ Ab were incubated with SN or standard (0.5 μL of bead with 50 μL of capture diluent per tube) for 1 h at room temperature (RT) in the dark. Then, an anti IFNγ Abs coupled to PE (0.5 μL of Abs with 50 μL of PE detection per tube) was added for 2 h at RT in the dark. Then, tubes were washed with 1 mL of wash buffer and centrifuged at 600× *g* for 5 min. After discarding SN, the acquisition was carried out on Canto II (at least 500 events/cytokine) and analysis using FCAP Array software. The use of standard allowed determining the relationship between the PE intensity and the concentration of cytokines, allowing us then to calculate the concentration of IFNγ in the SN.

### 2.8. Cross-Priming of Antigen (A2L) Specific CD8 T Cells Using ADDomers

#### 2.8.1. Conditions of Cross-Priming

HLA-A2+ PBMCs were thawed in complete medium RPMI 20% FCS, centrifuged at 400× *g* for 8 min, then resuspended in complete medium RPMI 10% FCS. Cells were plated at 1.10^6^ cells/mL in a 24 well plate and cultured for 7 days at 37 °C 5% CO_2_ in different conditions: control, or immunodominant HLA-A2 restricted peptide MelA_26–35_ at 10 μM, or ADD Ø (without peptide)/ADDomer A2L (containing MelA_26–35_ peptide) at 0.15 μM. All these conditions were performed with or without a mixture of TLR-L (mix Poly IC (TLR3L) 30 μg/ mL, R848 (TLR7/8L) 1 μg/mL and CpGA (TLR9L) 1 μM). At day 7, cells were harvested, washed, numerated and resuspended at 1.10^6^ cells/mL. Then, 200,000 cells were taken from each condition for dextramer staining (to evaluate the proportion of anti-MelA_26–35_ CD8+ T cells); the remaining cells were restimulated in the same conditions as at day 0. At day 13, the same protocol was performed. At day 20, all cells were collected for dextramer staining and analyzed by flow cytometry using a Canto II.

#### 2.8.2. Determination of Anti-MelA_26–35_ CD8+ T Cells by Dextramer Staining

Dextramer staining was performed at day 0 on 2.10^6^ cells, and at day 7, 13 and 20 on 200,000 cells harvested from the cultures. Cells were washed with 2 mL PBS 2% FCS, resuspended in 100 μL PBS 2% FCS, and stained for 20 min at RT with 5 μL of MHC dextramer MelA_26–35_ A*0201/ ELAGIGILTV (PE) from Immudex^®^ according to the manufacturer’s instructions. Cells were then stained with 5 μL of anti-human CD3 (labeled with the fluorophore PC7) and 5 μL of anti-human CD8 (labeled with the fluorophore APC) both from Beckman Coulter^®^ for 15 min according to the manufacturer’s instructions. Cells were washed with 2 mL PBS 2% FCS, resuspended in 300 μL of FACS lysing solution 1× and stored at 4 °C until acquisition. The proportion of dextramer+ CD8+ T cells together with the intensity of the dextramer labeling were analyzed using a Canto II and DIVA 6.0 software from BD.

#### 2.8.3. Amplification of Antigen Specific CD8+ T Cells

Calculation of absolute numbers of CD8+ T cells was carried out based on the percentage of CD8. Cells number in wells and the percentage of dextramer+ within CD8+ T cells allowed us then to calculate the absolute number of dextramer+ CD8+ T cells. The amplification of antigen specific CD8+ T cells was calculated based on the ratio of absolute numbers of dextramer+ CD8+ T cells at day X/day 0.

#### 2.8.4. Determination of the Affinity of Antigen Specific CD8+ T Cells

The affinity of the amplified dextramer+ CD8+ T cells was measured thanks to the Mean Fluorescence Intensity (MFI) of the dextramer labelling.

### 2.9. Detargeting Retargeting Experiments

#### 2.9.1. Evaluation of the Interactions between the ADDomers and CLR by Surface Plasmon Resonance

DC-SIGN Biot-ECD and MGL Biot-ECD, used in these SPR measurements, were overexpressed and purified according to previously published protocol [17,18]. A surface plasmon resonance experiment was performed on a T200 instrument. Streptavidin (Ref.: S4762; SIGMA-ALDRICH) diluted at 100 µg/mL in 10 mM NaOAc pH 4 was immobilized on sensor chips sensor chip S Serie CM3 (Cytiva, France). Biotinylated version of DC-SIGN ECD and MGL ECD (called, respectively, DC-SIGN Biot-ECD and MGL Biot-ECD) were used for their capture onto the streptavidin functionalized surfaces. These Biot-ECD version of both CLRs results from a site directed N-terminal biotinylation that allow a uniform orientation of the CLRs ECD on the surface mimicking the natural presentation at the cell surface. This site specific biotinylation on the N-termini are performed in house thanks to a sortagging procedure previously described in Achilli et al. [19]. DC-SIGN Biot-ECD and MGL Biot-ECD were diluted, respectively, at 0.5 µg/mL and 1 µg/mL in HBS P+ (Cytiva), injected at 5 µL/min until a capture level around 1250 RU (1264.4 RU for DC-SIGN Biot-ECD and 1254.0 RU for MGL Biot-ECD) was achieved. For interaction measurements, ADD KGE and ADD KGE RBD were prepared in HEPES 10 mM pH 7.4, NaCl 150 mM, CaCl_2_ 4 mM, 0.05% P20. They were injected at concentrations ranging from 0.3 nM to 136.5 nM over DC-SIGN Biot-ECD or a MGL Biot-ECD oriented surface using HBS P+ buffer supplemented with 4 mM CaCl_2_ as running buffer at 100 µL/min. RBD-SC was in HEPES 10 mM pH 7.4, NaCl 150 mM, CaCl_2_ 4 mM, 0.05% P20) and was injected over a DC-SIGN Biot-ECD or MGL Biot-ECD oriented surface at a concentration ranging from 56 nM to 28.75 µM using HBS P+ buffer supplemented with 4 mM CaCl_2_ at 100 µL/min. Streptavidin flow cell surface was used as reference for correction of the binding response. Regeneration of the surfaces was achieved by 50 mM EDTA, pH 8. Binding curves were analyzed using Biacore T200 Evaluation Software 3.2.1 (GE Healthcare) and data were fit using Steady State Affinity model. For ADD KGE RBD, only sensorgram comprised between 8.5 nM down to 0.2 nM were considered for determining the *K*_Dapp_ for DC-SIGN. In this range of concentrations, a nice saturation curve could be observed in steady state analysis. The higher concentration tested from 8.5 nM up to 136 nM still contributes to higher binding to the surface but in a non-specific mode (linear increase) and thus were not considered in the analysis.

#### 2.9.2. De-Targeting Evaluation Using ADD KGE versus ADD RGD on A549 Cell Line

A549 cells were grown as monolayers in RPMI-1640 Glutamax (Gibco^®^, France) containing 10% fetal bovine serum (Gibco^®^), 5,000 U/100 mL of penicillin-streptomycin (Gibco^®^, France) at 37 °C in 5% CO_2_ using T75 flasks, or culture dishes as specified.

#### 2.9.3. ADD KGE and RGD Labeling with AF 488

ADD KGE and ADD RGD were first incubated with the fluorochrome NHS-Alexa 488 (ThermoFisher, France) overnight at 25 °C under agitation on a ThermoMixer (Eppendorf, France) at 300 rpm. The NHS moiety forms a covalent bond with the ADDomers’ lysins terminal NH_2_ residues.

#### 2.9.4. ADD KGE and ADD RGD Internalization by A549 Evaluated by Flow Cytometry

A549 cells were seeded at 5.10^4^ cells per cm^2^ in a nunclon delta surface 4 wells of 1.9 cm^2^ multidish (ThermoFisher Scientific, France) and incubated overnight at 37 °C, 5% CO_2_. The next day medium was replaced with 300 µL of fresh culture medium containing 1µg of each ADDomer (ADD KGE AF488 or ADD RGD AF488) and cells were further incubated for 1 h at 37 °C, 5% CO_2_. Cells were then washed in PBS 1× and were detached by the addition of 100 µL of trypsin in each well for 5 min at 37 °C. This step aims at removing the ADDomers fixed on the cells’ surfaces while not affecting the ADDomers internalized by the cells. 900 µL of fresh medium were added and the suspension transferred into 1.5 mL tubes. Cells were pelleted by centrifugation, washed in 500 µL of PBS 1×, then resuspended in 1 mL of PBS, 1% PFA for cell fixation. Analysis was performed directly after fixation by flow cytometry (M4D platform, VYB, Miltenyi biotech, Paris, France). The time between ADDomer’s removal and the analysis was estimated to be 20 min.

#### 2.9.5. ADD KGE and ADD RGD Internalization by A549 Evaluated by Time-Lapse Confocal Microscopy

A549 cells were seeded at 5.10^4^ cells per cm^2^ in a coverglass bottom 4 compartments 35 mm dish (Cellview™ Greiner, Paris, France) and incubated overnight at 37 °C, 5% CO_2_. The next day, medium was replaced with 300 µL of fresh culture medium containing 10 µg of each ADDomer (ADD KGE AF488 or ADD RGD AF488) and cells were further incubated for 1 h at 37 °C, 5% CO_2_. The dish was then immediately imaged using a spinning disk confocal microscope with a 100× 1.49NA apochromatic oil immersion objective. Imaging was performed using an iXon Ultra EMCCD (16bits per pixel, IQ bioimaging software, Andor) for 1 h, acquired every 15 s for AF488 fluorescence, and every 150 s for transmission using differential interference contrast (M4D platform, IX83 Olympus; CSU-XI Yokogawa; Okolab incubation chamber kept at 28 °C, 5% CO_2_). Both conditions were imaged in parallel. Analysis was generated using Imaris (Bitplane, Paris, France) and Fiji open-source software [20]. The time between ADDomer’s removal and the analysis was estimated to be 1 or 2 min.

## 3. Results

### 3.1. Human Melanoma Epitope A2L Can Be Displayed on the ADDomer Surface by Genetic Insertion

**Figure 1:**
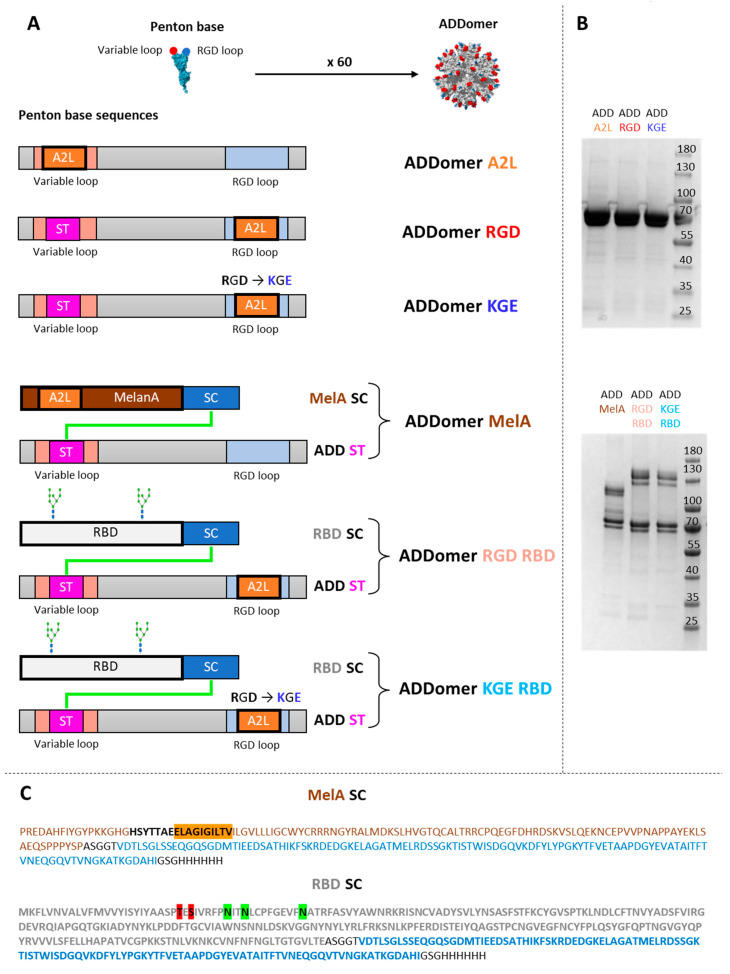
**Design and characterization of the different ADDomers**. (**A**) Diagram showing the insertion of the A2L epitope (A2L in orange) and SpyTag (ST in pink) in the 2 loops of the ADDomer monomer. SpyTag can make an isopeptidic bond with SpyCatcher (SC in blue) fused to the RBD protein (in grey) or the MelanA protein (MelA in brown). (**B**) SDS-PAGE profiles of reduced and boiled samples of the different ADDomers showing the apparition of a higher MW covalent adduct for the ADD-MelA (ADD-ST/MelA-SC), ADD KGE RBD (ADD-KGE/RBD-SC) and ADD RGD RBD (ADD-RGD/RBD-SC). (**C**) Sequences of the MelA-SC and RBD-SC. In the MelA-SC sequence, the MelanA tumor antigen is colored in brown (A2L epitope is highlighted in orange), SpyCatcher is colored in blue and additional linkers and histidine tag are colored in black. In the RBD-SC sequence, the Spike Receptor Binding Domain of SARS-CoV-2 is colored in grey (N and O-glycosites are highlighted, respectively, in green and red), SpyCatcher is colored in blue and additional linkers and histidine tag are colored in black.

The ADDomer is a non-infectious nanoparticle formed of 12 bricks of the homo-pentameric penton base from the human adenovirus type 3. Two exposed loops can be used for the insertion of epitopes, namely the variable loop (VL) and the RGD loop (RGD-L) (Figure 1A). A sequence containing the A2L epitope from the tumor associated antigen MelanA (MelanA_26–35_) was genetically inserted into the VL. This epitope has been chosen to initiate a CD8 + T lymphocyte immune response via the epitope A2L presented to the CD8 by DCs. Due to the spontaneous homo-oligomerization of the 12-pentameric penton bases, 60 copies of each epitope are exposed on the surface of the ADDomer particle named ADDomer-A2L (ADD-A2L) (Figure 1A). The ADD-A2L was successfully produced in a baculovirus expression system and purified in two steps (Figure 1B, upper panel).

### 3.2. The Large Human Tumor Associated Antigen MelanA Can Be Displayed on the ADDomer’s Surface Using the SpyTag/SpyCatcher System

In order to get rid of the allelic restriction and to elicit a multi-specific anti-tumor response, the display of large antigens rather than small epitopes on the ADDomer surface is desirable. To do this, an ADDomer displaying the SpyTag peptide (ADD-ST) on its surface (Figure 1A,C) was used to spontaneously establish an isopeptidic bond with a SpyCatcher (SC) fused to the MelanA protein containing the A2L epitope. The MelanA protein was genetically fused at the SpyCatcher N-terminus (MelanA-SC) and a 5 amino acid linker was used to separate the MelanA and SpyCatcher sequences. The SpyCacther was extended with a histidine tag on the C-terminus (Figure 1C). ADD-ST and MelanA-SC were produced separately in insect cells using the baculovirus expression system.

The covalent binding of MelanA-SC to ADD-ST was assessed by SDS-PAGE. As expected, a higher molecular weight corresponding to an adduct of the MelanA-SC bound to the ADD-ST monomer was clearly seen (Figure 1B, lower panel). This ADDomer displaying the MelanA antigen was named ADD MelA.

### 3.3. ADD A2L and ADD MelA Are Differently Internalized by DC Subsets

To investigate the ability of human DC subsets to fix by adhesion at the cell surface, or internalize ADDomers, fluorescent ADD A2L and ADD MelA labeled with Alexa Fluor 647 (AF 647) were incubated for 1h with PBMC containing the three major human DC subsets: plasmacytoid DC (pDC), conventional DC1 (cDC1) and conventional DC2 (cDC2). Plasmacytoid DC are specialized to sense and respond to viruses, whereas conventional DC are more specialized in cross presentation via MHC class I to activate CD8^+^ T cells [21]. Cells were then either washed and directly analyzed by flow cytometry or coated on microscope slides and stained with specific DC subsets markers. No “cell-scraping” treatment was performed, meaning that the flow cytometry experiment would reveal ADDomers fixed on the cell surfaces as well as ADDomers internalized. Confocal microscopy was used to decipher the internalization of ADDomers by the DC subsets.

**Figure 2:**
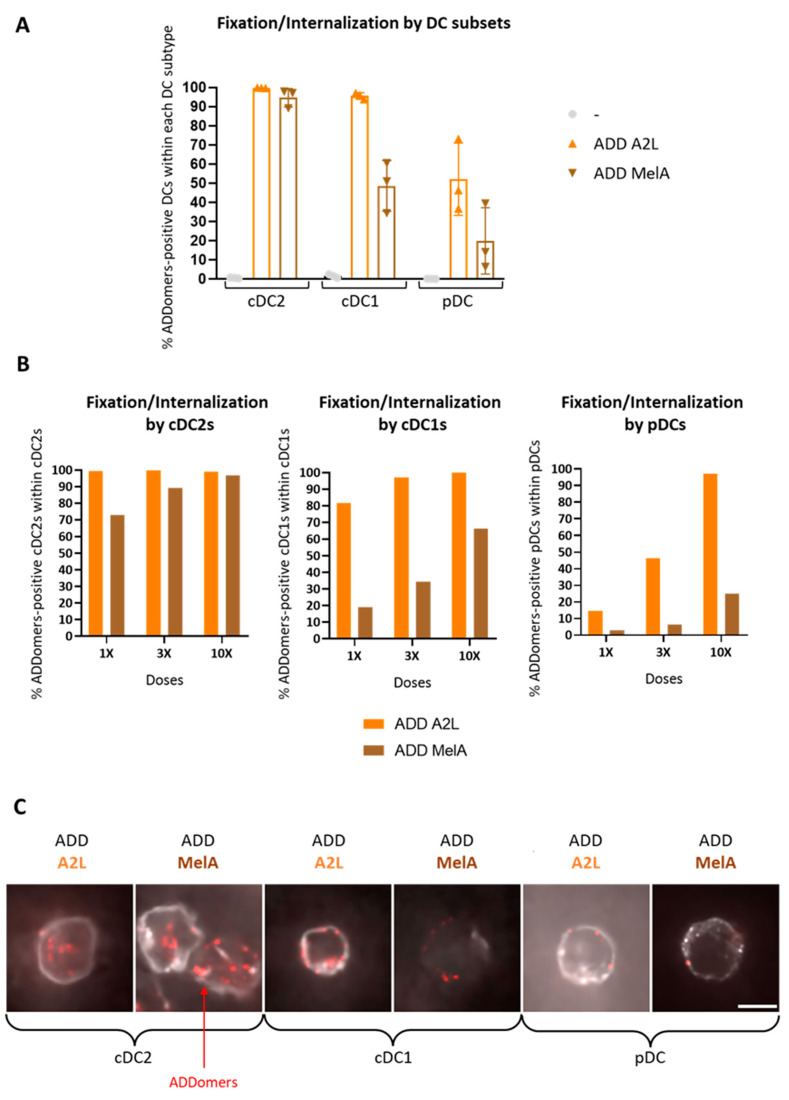
**DC subsets efficiently fix and internalize ADDomers.** PBMC (**A**,**B**) or PanDC (**C**) were incubated with different doses (1×, 3×, 10×) of fluorescent A2L/MelA-ADDomer for 1 h (**A**,**B**) or 2 h (**C**). The fixation and internalization of ADDomer by DC subsets were then evaluated by flow cytometry and confocal microscopy. (**A**) Percentage of fixation of ADDomer (3×) by each DC subset (*n* = 3 donors). (**B**) Dose response of ADDomer fixation by DC subsets (*n* = 1 donor). (**C**) Confocal imaging of ADDomer internalization by DC subsets. ADDomers are colored in red and the specific underlying BDCA1, BDCA2 or BDCA3 labeling is shown in gray. Scale bar is 5 microns and images are 18 × 18 microns.

Analysis by flow cytometry shows that both ADDomers are fixed/internalized by all DCs but revealed distinct efficiency between subsets. Whereas cDC2s strongly fixed/internalized ADDomers, cDC1s were a little less efficient, and pDCs even less (Figure 2A, Supplementary Figure S3). There is also a dose response effect for both ADDomers suggesting specific behavior of the ADDomers (Figure 2B). Interestingly, ADD MelA is less fixed/internalized than ADD A2L by all DC subsets. Such a decrease in fixation can be caused by the shielding of ADDomer’s integrin binding motif (RGD motif in the RGD loop) by the MelanA-SC on the ADDomer’s surface, since this motif has been shown to promote internalization in cells [22,23]. Confocal microscopy images further revealed that cDC2s massively engulfed ADDomers, whereas fluorescent ADDomers looked less internalized and more localized on the surface of cDC1s. Very few ADDomers looked localized on the surface of pDCs (Figure 2C). These observations suggest that ADDomers are not only fixed but also internalized by the DC subsets cDC2s and cDC1s.

### 3.4. ADDomer Triggers a Strong Activation of All DC Subsets Together with Cytokine Secretion

We then wanted to investigate the impact of ADDomer on DC subsets, especially their ability to activate them. Thus, PBMC (containing all DC subsets) were cultured either with a mixture of TLR ligands (TLR3-L, TLR7/8-L, TLR9-L) that stimulate cDC1s, cDC2s and pDCs, or peptide MelA_26–35_ (10 μM), ADD Ø (i.e., ADDomer which does not display any epitope) or ADD A2L (1 μM). TLR (Toll-Like-Receptors) are pathogen-recognition receptors that dimerize after the binding of their ligand (TLR-L) and become activated. Through the signaling pathway, they will stimulate the DCs, leading to a higher cross presentation, triggering a better cross priming.

**Figure 3:**
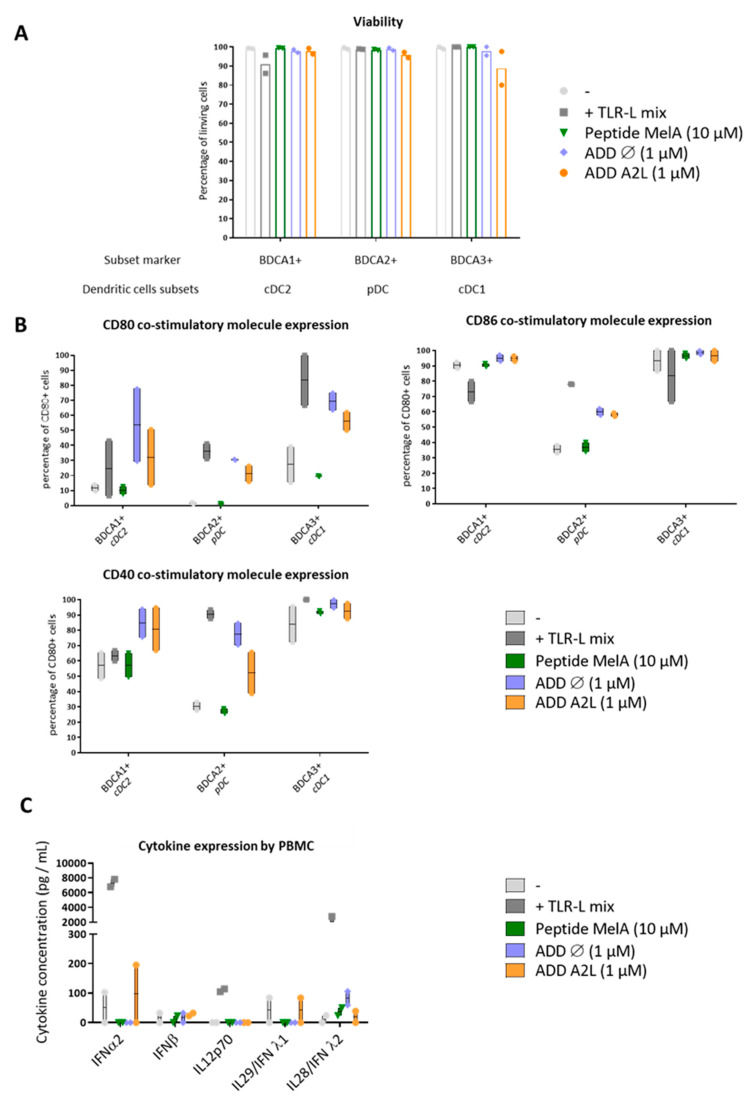
**ADDomer triggers a strong activation of DC subsets together with cytokine secretion.** PBMCs were cultured for 24 h in control conditions (−), or with either a mixture of TLR-L (Poly IC, R848, CpGA), MelA_26–35_ peptide (10 μM), empty ADDomer (ADD Ø) or displaying A2L (ADD A2L) both at 1 μM. The viability, activation and cytokine secretion by DC subsets were then evaluated. (**A**) Impact of ADDomer on DCs’ viability assessed by measuring the percentage of alive cDC2s (BDCA1+), cDC1s (BDCA3+) and pDCs (BDCA2+) (*n* = 2 donors). (**B**) Impact of ADDomer on DCs’ activation depicted by evaluating the percentage of CD40+, CD80+ and CD86+ DC subsets (*n* = 2 donors). (**C**) Cytokine secretion measured in the culture supernatants by Luminex (*n* = 2 donors).

Within alive CD45+ cells, DCs were defined as HLA-DR+ and the absence of Lin. cDC2s were gated as CD11c+ BDCA1+. pDCs were defined as CD11c negative and BDCA2+, while cDC1s were depicted as CD11c+/BDCA3+. The viability of DC subsets was first assessed using live&dead labeling. We observed that both ADDomers were not toxic for DCs within PBMCs, as the percentage of living cells was above 90% whatever the conditions (Figure 3A). The activation status of DC subsets was further depicted by investigating the expression of costimulatory molecules (CD40, CD80 and CD86) by flow cytometry. As expected, the TLR-L mix (positive control) up-regulated CD80, CD40 and/or CD86 molecules on each DC subset compared to the control condition, demonstrating their proper activation. Peptide MelA_26–35_ did not activate DCs, as there was no significant up-regulation of the costimulatory molecules (Figure 3B). Noteworthy, both ADDomers (ADD Ø and ADD A2L) triggered a strong activation of BDCA1 (cDC2s), BDCA2 (pDCs) and BDCA3 (cDC1s) subsets, highlighted by the up-regulation of the three costimulatory molecules (from 13 to 100% depending on the donor and the DCs subset). This up-regulation is as high as in TLR-L condition or even higher. This observation reveals the potent adjuvant properties of the ADDomer itself as even the ADD Ø can activate the DC subsets (Figure 3B). Furthermore, even if TLR-L stimulation leads to huge levels of cytokines secretion, ADD Ø and A2L triggered low level of IL28/IFNλ2 secretion (maximum of 106 pg/mL for 1 donor compared to 24 pg/mL for the negative control) or low level of IFNα2 expression (maximum of 195 pg/mL for 1 donor compared to 102 pg/mL for the negative control) and IL29/IFNλ1 (Figure 3C). These results suggest the weak but potent immune-stimulating potential of ADDomers on all DC subsets.

### 3.5. DCs Efficiently Cross-Present the A2L Epitope from ADDomer Displaying the Full MelanA Antigen

**Figure 4:**
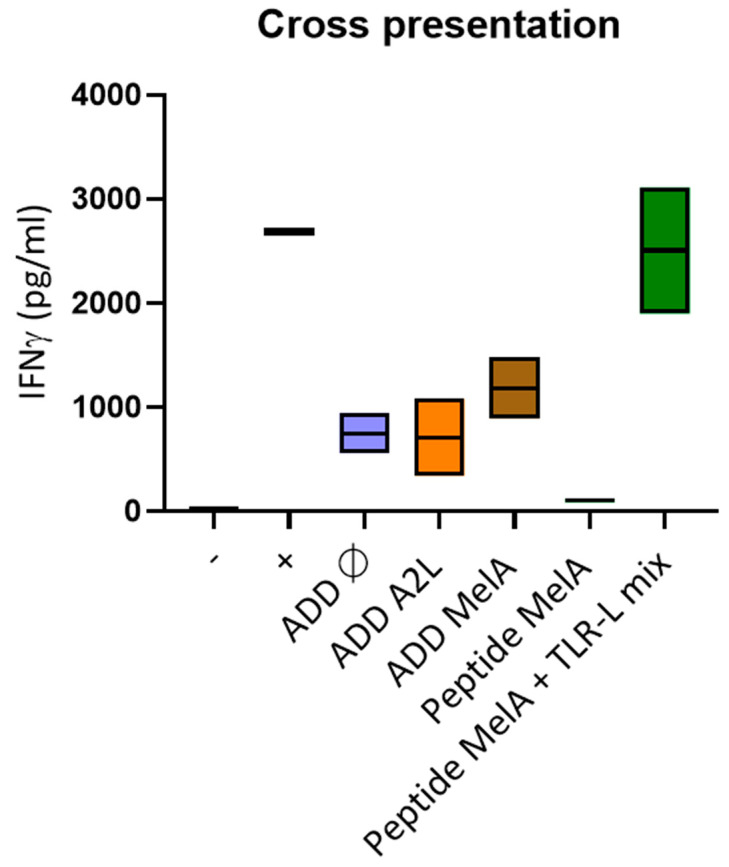
**DCs efficiently cross-present the A2L epitope from ADDomer displaying MelA.** Purified panDCs were incubated with ADD Ø, ADD A2L, ADD MelA, Peptide MelA and Peptide MelA + TLR-L for 1 h and cocultured 20 h with MelA_26–35_ specific CD8+ T cells. IFNg secretion was then evaluated by CBA. T cells were also cultured alone (−) or with PMA/iono (+) (*n* = 2 donors).

To determine if the ADDomer could be cross-presented by human DCs, panDCs purified from HLA-A2+ PBMC (mix of cDC2s, cDC1s and pDCs) were pulsed with ADD Ø, ADD A2L, ADD MelA or peptide MelA_26–35_ as the positive control. Pulsed panDCs were then co-cultured with HLA-A2 restricted T cells containing pre-amplified anti-MelA_26–35_ CD8+ T cells. The production of IFNγ was analyzed by CBA (Cytometric Bead Array). The success of the cross presentation was revealed by the ability of T cells to produce IFNγ upon recognition of the complex peptide/HLA-A2 complex presented by the panDCs. The results show that ADD MelA seems to lead to a higher IFNγ production than ADD Ø and ADD A2L (Figure 4). This suggests that ADD MelA is cross-presented by dendritic cells.

### 3.6. ADDomer Displaying A2L Epitope Cross-Prime High Affinity Peptide-Specific CD8+ T Cells

Based on the previously observed ability of DC to fix, internalize and cross-present ADDomers, we further explored whether DCs could cross-prime peptide-specific CD8+ T cells from ADDomers displaying epitopes. To assess this point, PBMCs from 3 HLA-A2+ donors (containing < 0.03% of naïve MelA_26–35_–specific CD8+ T cells) were incubated in control conditions (negative control), or with peptide MelA_26–35_ (positive control), or with ADD Ø or with ADD A2L (0.15 μM). These conditions were performed in the presence or absence of a mixture of TLR ligands (TLR3-L, TLR7/8-L, TLR9-L) that stimulate cDC1s, cDC2s and pDCs. As described before TLR will stimulate the DCs leading to a higher cross presentation, triggering a better cross priming. Dextramer staining was performed to measure the percentage of anti-MelA_26–35_ dextramer+ CD8+ T cells at days 0, 7, 13 and 20 (Supplementary Figure S4), the relative amplification of Ag-specific T cells as well as the affinity of the amplified dextramer+ CD8+ T cells.

**Figure 5:**
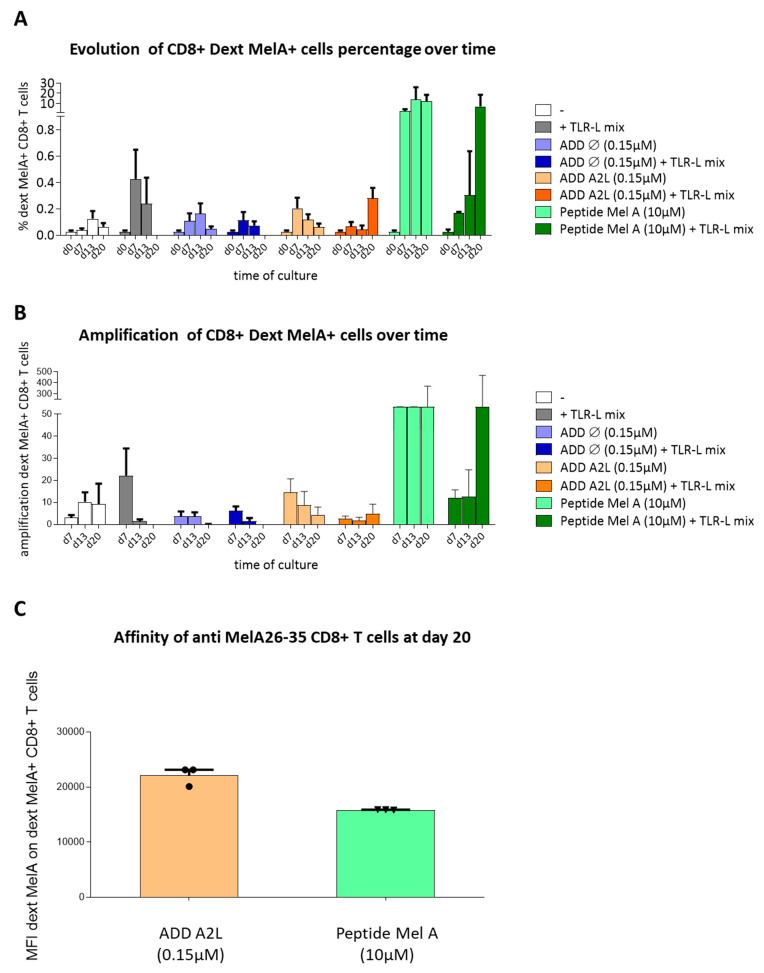
**ADDomer displaying A2L epitope efficiently cross-prime peptide-specific CD8+ T cells dotted with a high affinity.** PBMC were cultured in control conditions or with empty ADDomer (ADD Ø) or ADD A2L in the presence or not of a mixture of TLR-L. Cultures were restimulated every 7 days for 3 weeks. The elicitation of MelA-specific CD8 T cells was then evaluated by dextramer staining. (**A**) Evolution of Dext MelA+ CD8 T cells over time in the different conditions (*n* = 3 donors). (**B**) Amplification of Dext MelA+ CD8+ T cells over time. The amplification of antigen-specific CD8+ T cells was calculated based on the ratio of absolute numbers of dextramer+ CD8+ T cells at day X/day 0 (*n* = 3 donors). (**C**) Affinity of Dext MelA+ CD8+ T cells at day 20 (*n* = 3 donors).

As expected, there is no triggering of anti-MelA_26–35_ CD8+ T cells in control conditions until D20 (percentage below 0.1%), whereas an amplification of dextramer+ CD8+ T cells (from 0.09% up to 22%) is observed for the 3 donors with the peptide MelA_26–35_ (positive control) with and without the TLR-L mix. Concerning the ADD Ø, we noticed negligible or no elicitation (percentage below 0.4% of dextramer+ CD8+ T cells for the 3 donors) (Figure 5A), revealing that the ADDomer itself does not trigger anti-MelA_26–35_ CD8+ T cells, even though it strongly activated DCs.

Notably, ADD A2L was able to trigger dextramer+ CD8 T cells optimally at day 7 in the absence of TLR-L, and at day 20 in the presence of TLR-L mix (maximum 0.359% of dextramer+ CD8+ T cells at D20). Therefore, the results suggest that TLR-L mix is required to optimally stimulate the DCs for triggering anti-MelA_26–35_ CD8+ T cells from ADD A2L (Figure 5A).

Furthermore, the amplification of anti-MelA_26–35_ CD8+ T cells was calculated as the ratio of absolute numbers of antigen specific CD8+ T cells from day 0 (Figure 5B). For the negative control, no significant amplification of anti-MelA_26–35_ CD8+ T cells was observed, whereas, for the peptide MelA_26–35_ in the absence of TLR-L mix (positive control), an amplification at day 20 was noticed. For the ADD Ø, a negligible amplification (maximum of 7.51 without TLR mix and 10.08 with the TLR mix at day 7) was detected. Notably, concerning the ADD A2L, there was no obvious amplification with TLR mix (maximum of 9.20). However, when stimulated without TLR mix we observed an amplification of 23.41 at day 7 (Figure 5B).

Finally, the affinity of the anti-MelA_26–35_ CD8+ T cells could be defined by using the MFI of the dextramer labeling, knowing that a higher MFI corresponds to a better affinity (Figure 5C). By comparing the affinity of the anti-MelA_26–35_ CD8+ T cells, it appears that the one triggered by ADD A2L is higher than the one elicited by the peptide MelA_26–35_ itself. Indeed, the MFI for the 3 donors tested tends to be 33% higher for the ADD A2L (~20,000) than for the peptide MelA_26–35_ (~15,000). This observation highlights the ability of ADD A2L to trigger high affinity anti-MelA_26–35_ CD8+ T cells (Figure 5C).

### 3.7. De-Targeting and Re-Targeting ADDomers Specifically to DC Subsets

ADDomers present a very specific integrin binding motif (RGD motif in the RGD loop [22,23]), allowing them to be internalized by most cells [22,23]. With the aim of limiting off-target effects, we mutated the RGD motif into KGE. To direct the ADDomers to dendritic cells, the Spike Receptor Binding Domain of SARS-CoV-2 (from K300 to E554) fused to the SpyCatcher (RBD-SC) was displayed on the ADDomer’s surface via the SpyTag/SpyCatcher system [15]. The A2L epitope was then moved from the VL to the RGD loop next to the RGD/KGE motif (Figure 1A). RBD-SC is secreted by insect cells during its production in baculovirus expression system and is therefore glycosylated (mostly poly-mannoses [24,25]). Indeed, 3 putative N-glycosites (N331, N334 and N343 highlighted in green) and 2 putative O-glycosites (T323 and S325 highlighted in red) are present in the RBD-SC sequence [26] (Figure 1C). The glycosylation of RBD-SC has already been assessed in Chevillard et al., 2022 [15]. These N-Glycan motifs can be recognized by different C-type lectin receptors (CLRs) present on the dendritic cell surface [27] as a function of the nature of the N-Glycan and its level of processing. Indeed, high-mannose or hybrid type N-glycan, which both expose terminal mannose residue, will be recognized by CLRs such as DC-SIGN, while MGL may binds either Hybrid or Complex-type N-Glycan, exposing terminal galactose. The covalent binding of RBD-SC to ADD-ST was assessed by SDS-PAGE. As expected, an adduct of a higher molecular weight corresponding to the RBD-SC bound to the ADD KGE or ADD RGD monomer (both bearing the ST motif) was clearly seen (Figure 1B, lower panel).

### 3.8. Functional De-Targeting of ADD KGE Compared to ADD RGD on A549 Cells

**Figure 6:**
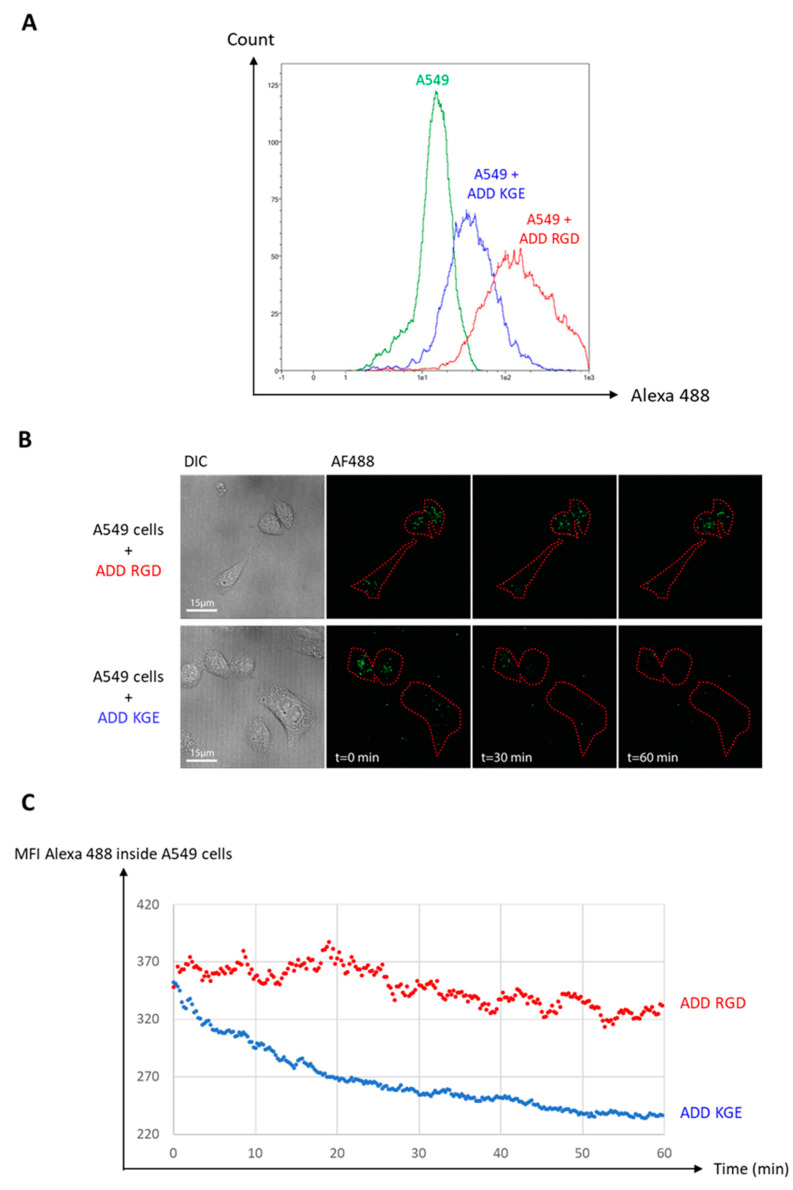
**ADD KGE and ADD RGD internalization by lung epithelial cell line A549.** (**A**) ADD KGE AF 488 and ADD RGD AF 488 internalization by lung epithelial cell line A549 evaluated by flow cytometry at t = 20 min. (**B**) Time lapse imaging by confocal microscopy of ADD KGE AF 488 and ADD RGD AF 488 internalization by lung epithelial cell line A549. (**C**) Mean Fluorescence Intensity (MFI) of ADD KGE AF 488 and ADD RGD AF 488 within cells over time.

To assess ADDomer’s de-targeting via the KGE motif, A549 adherent cells were incubated for 1h with ADD KGE and ADD RGD, both labeled with identic quantities of AF 488. Both ADDomer’s internalization inside the A549 cells was evaluated by flow cytometry, and microscopy. A549 adherent cells (lung epithelium cell line) were used as a model of epithelial cells which can participate in off-target effects. For the flow cytometry analysis, after incubation with the fluorescent ADDomers, cells were treated with trypsin in order to remove the ADDomers fixed on cell surfaces while not affecting the ADDomers internalized by the cells. The total duration of this treatment was 20 min, as described in materials and methods. Thanks to the trypsin treatment, the fluorescence intensities represented in Figure 6A correspond to ADD KGE and ADD RGD, which have been internalized. The fluorescence intensities at t = 20 min (1 h of incubation + 20 min of treatment for flow cytometry analysis) corresponding to the ADDomers (represented in blue for ADD KGE and in red for ADD RGD) (are both higher than the one corresponding to the negative control (in green), strongly suggesting they have been both internalized. Moreover, as both ADDomers are stoichiometrically labeled with AF 488 (data not shown), we can infare that the difference in fluorescence intensities between the 2 ADDomers corresponds to their respective internalization levels, suggesting internalization levels by A549 are higher for ADD RGD than for ADD KGE reflecting a partial de-targeting of this latter.

For the microscopy analysis, cells were incubated for 1h with the fluorescent ADDomers at 37 °C, 5% CO_2_, and then monitored for another hour using a spinning disk confocal microscope. The Figure 6B shows that at t = 0 (1 h of incubation) both ADDomers (represented in green spots) are localized within the cells (underlined in red). At t = 60 min (2 h of incubation) however, ADD RGD are still clearly visible within the cells whereas ADD KGE are much less present. The quantitative analysis of mean fluorescent intensities in the cells (red regions of interest) in both cases (Figure 6C) shows that ADD RGD associated fluorescence is almost retained while ADD KGE fluorescence is lost after 2 h. This suggests that ADD KGE but not ADD RGB is degraded during this lap time. As both moieties are internalized, this latter finding suggests that ADD RGD and ADD KGE might use different internalization mechanisms.

### 3.9. Assessment of ADDomer Targeting to C-Type Lectin Receptors (CLRs) by SPR

**Figure 7:**
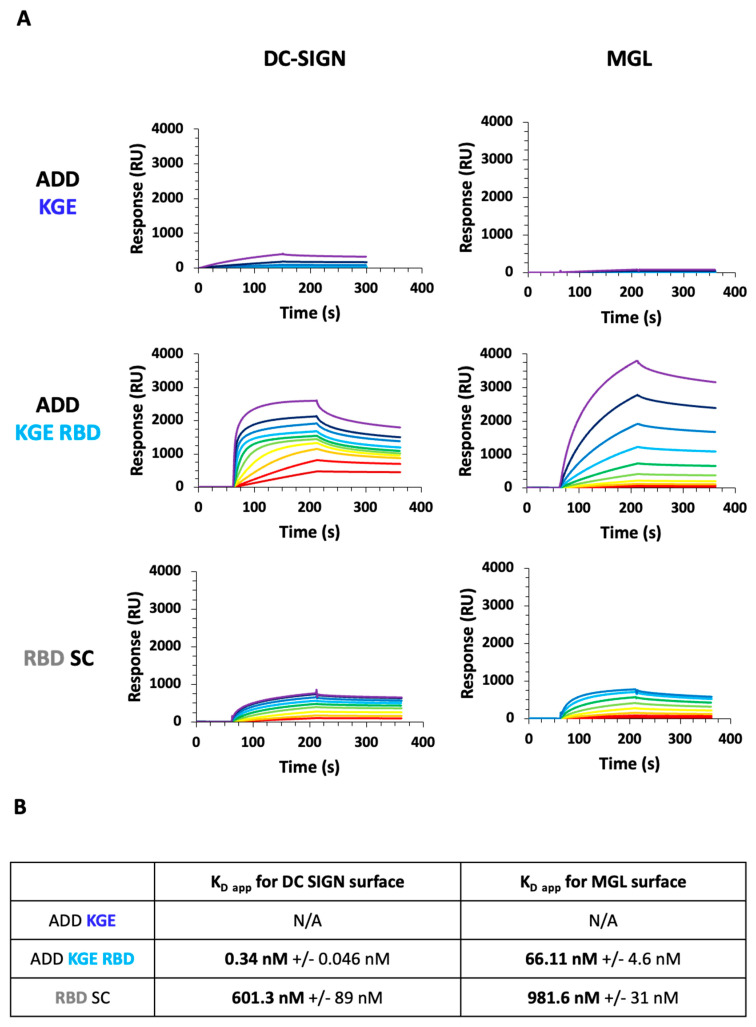
**ADD KGE, ADD KGE RBD and RBD-SC binding evaluation on DC-SIGN and MGL receptors by surface plasmon resonance.** (**A**) Sensorgrams of the interaction of the ADD-KGE constructs and RBD-SC onto DC-SIGN or MGL oriented surface. For ADD KGE and ADD KGE RBD, injected concentrations range from 136 nM to 0.2 nM by cascade dilution by a factor of 2. For RBD SC, concentration used range from 14.4 µM down to 56 nM by cascade dilution by 2. (**B**) Table of apparent K_D_ calculated using steady state analysis in the BIAEval software.

DCs especially cDC2s express C-type lectin receptors, such as DC-SIGN and MGL. ADD KGE, ADD KGE RBD and RBD-SC interaction with DC-SIGN and MGL receptors (tetrameric and trimeric protein, respectively) was measured by SPR. To do so, these ADDomers were injected over streptavidin surfaces functionalized with the extracellular domains (ECD) of DC-SIGN or MGL receptors modified with a biotin at their N-terminus (respectively called DC-SIGN Biot-ECD and MGL Biot-ECD). This specific N-terminal biotinylation allows the surface functionalization in an oriented manner, thus mimicking the membrane orientation of the DC-SIGN ECD and MGL-ECD. This allows an optimal access to the carbohydrates recognition domain (CRD)s of these multimeric receptors [28]. These 2 receptors can recognize glycosylation patterns [29,30] present on the RBD-SC. For ADD KGE, the sensorgrams revealed that the interaction is negligeable and thus no relevant: the K_D app_ could therefore not be determined (Figure 7). On the contrary, the addition of RBD to ADD KGE (ADD KGE RBD) resulted in very strong and reliable interactions for both lectin receptors. However, a strong preference for DC-SIGN over MGL have been observed with respective K_Dapp_ of 0.34 ± 0.005 nM and 66.1 ± 4.6 nM, respectively.

Finally, the RBD-SC alone shows significant interactions with the two receptors and like with ADD KGE RBD the interaction is stronger for DC-SIGN (K_Dapp_ = 601 ± 90 nM), than for MGL (K_Dapp_ = 982 ± 31 nM) (Figure 7). When comparing, for both lectins, the K_Dapp_ of the ADD KGE RBD with the ones of the single RBD-SC, a tremendous increase in affinity by a factor of about 1800 times was observed towards DC-SIGN and of “only” 15 times for MGL. This underlines the great enhancement of avidity, and thus targeting efficiency, that can be achieved thanks to the avidity generated by the multivalent RBD presentation within the ADD context. Moreover, the higher interaction increase observed with DC-SIGN compared to MGL, suggests that the RBD harbor more particularly N-Glycan specific to DC-SIGN.

### 3.10. ADD KGE/RGD and ADD KGE/RGD RBD Are Successfully Fixed/Internalized by DC Subsets but Differentially Depending on the Subset

**Figure 8:**
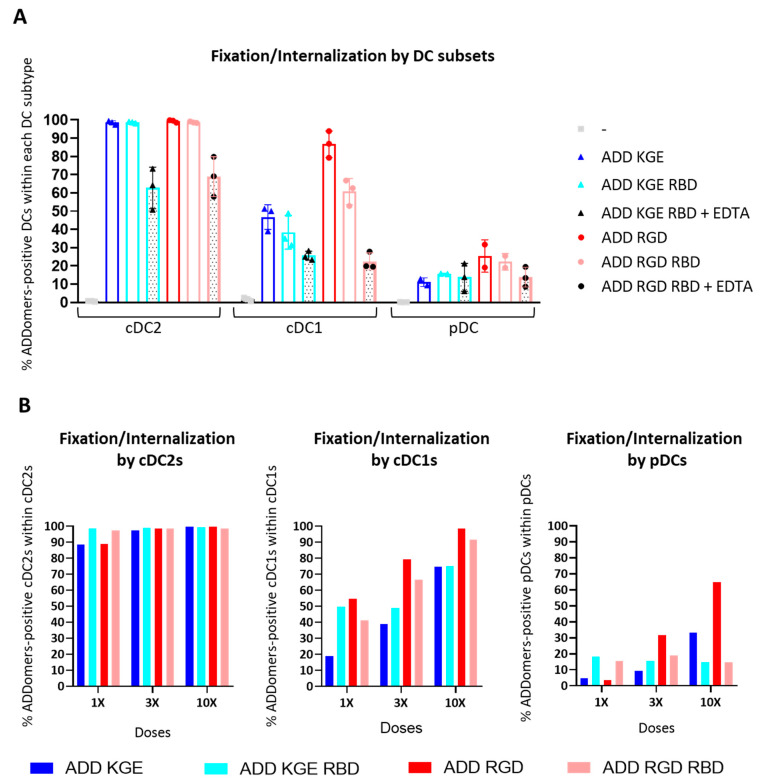
**ADD KGE/RGD (with or without RBD-SC and EDTA) fixation/internalization by DC subsets.** PBMC pre-incubated or not with EDTA were incubated with fluorescent KGE/RBD ADDomer for 1 h. The fixation of ADDomer by DC subsets was then evaluated by flow cytometry. (**A**) Fixation and internalization evaluation of ADD KGE/RGD (with or without RBD-SC and EDTA) (0.5 µM i.e., 3×) on DC subsets using flow cytometry (*n* = 3 donors). (**B**) Fixation and internalization evaluation of ADD KGE/RGD (with or without RBD-SC and EDTA) (0.15 µM i.e., 1× or 0.5 µM i.e., 3× or 1.5 µM i.e., 10×) on DC subsets using flow cytometry (*n* = 1 donor).

In addition, for ADD A2L and ADD MelA (Figure 2), we observed that all ADDomers are better fixed/internalized by cDC2s, than cDC1s than pDCs (Figure 8A,B, Supplementary Figure S5). Indeed, whereas cDC2s strongly fix/internalize ADDomers, cDC1s were a little less efficient, and pDCs even less. There is also a dose response effect for all ADDomers suggesting specific fixation/internalization (Figure 8B). Similar to ADD MelA, the addition of RBD-SC on both ADD KGE and ADD RGD seemed to decrease the fixation/internalization, even though it was less obvious than for ADD MelA and it varied between donors. This decrease in fixation/internalization can also be caused (for the ADD RGD versus ADD RGD RBD) by the shielding of ADDomer’s integrin binding motif [22,23] (RGD motif in the RGD loop) by the RBD-SC on the ADDomer’s surface. As mentioned for A549 cells, ADD KGE seems to be fixed/internalized by a non-RGD-dependent-integrin-mediated mechanism. One can imagine that this mechanism involves recognition of other molecular patterns present on the ADDomer’s surface which are also shielded by the RBD-SC.

### 3.11. ADD KGE/RGD RBD Fixation/Internalization by Dendritic Cells Might Involve Binding to a CLR

As SPR data showed a binding of glycosylated RBD to the CLRs DC-SIGN and MGL, CLR binding was investigated on DC subsets. To do this, ADDomers AF647 were incubated for 1h with PBMC containing DC subsets and internalization was evaluated by flow cytometry. The PBMC were previously incubated in the absence or in the presence of EDTA. EDTA chelates Ca^2+^ ions are involved in CLR-ligand binding as well as in the integrin mediated internalization. We hypothesized that an internalization decrease observed in the presence of EDTA for the ADD KGE RBD would suggest a non-integrin mediated internalization mechanism requiring Ca^2+^ is involved. According to the SPR data, this mechanism might involve CLRs such as DC-SIGN or MGL. This hypothesis was validated as a strong internalization decrease was noticed for ADD KGE RBD + EDTA compared to ADD KGE RBD (Figure 8A).

### 3.12. Only ADD RGD Is Cross-Presented by Dendritic Cells

**Figure 9:**
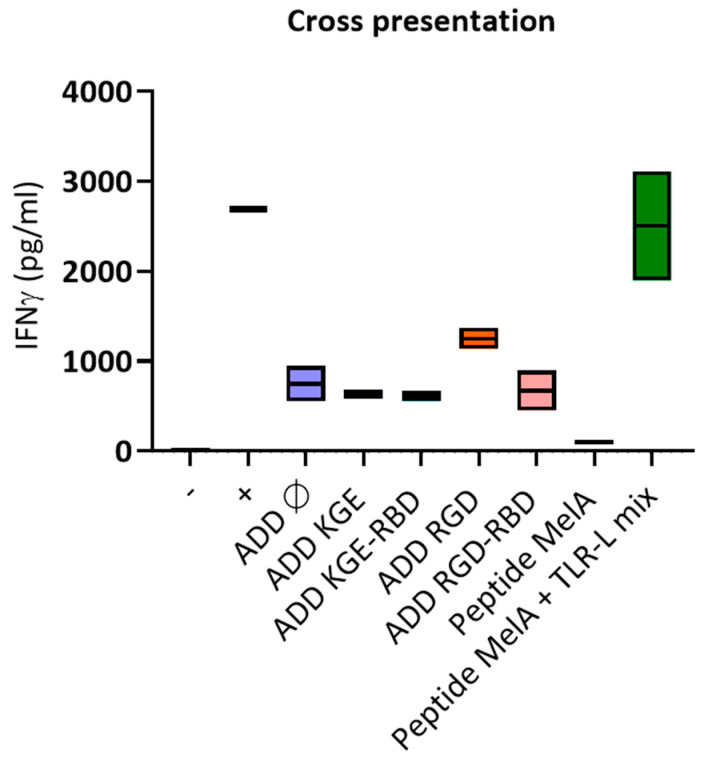
**DCs efficiently cross-present the A2L epitope only when displayed by ADD RGD.** Purified panDCs were incubated with ADD Ø, ADD KGE/RGD +/− RBD, Peptide MelA and Peptide MelA + TLR-L for 1 h and cocultured 20 h with MelA_26–35_ specific CD8+ T cells. IFNg secretion was then evaluated by CBA. T cells were also cultured alone (−) or with PMA/iono (+) (*n* = 2 donors).

A cross presentation experiment was performed similarly to the ADD MelA cross presentation experiment (Figure 4). The results show that only ADD RGD seems to lead to a higher IFNγ production than ADD Ø (Figure 9). This suggests that only ADD RGD is cross-presented by dendritic cells.

## 4. Discussion

In this work we assessed in vitro the immunogenicity on human PBMC from healthy donors of an “adenovirus-inspired” non-infectious VLP displaying a human melanoma tumor epitope or antigen and the impact of ADDomers on human DCs’ features. The VLP used results from the spontaneous assembly of twelve homopentameric penton bases from the human adenovirus of type 3 (HAdV-B3). The engineering of this 60-mer VLP named ADDomer (Adenovirus dodecamer) enabled an easy and rapid genetic insertion of short epitopes at two different locations of the particle surface. This vaccine platform displaying ovalbumin model antigen or epitopes was already proven to control melanoma tumor growth in mice bearing B16 OVA tumors [16].

MelanA (MelA) is a well-characterized melanoma differentiation antigen containing the immunodominant MHC class I A2L epitope (ELAGIGILTV). These MelanA and A2L antigen and epitopes are often used in preclinical and clinical studies evaluation of vaccine candidates against melanoma [31,32,33,34]. To bind the MHC-I proteins, class I epitopes must be processed by the proteasome into 8–10 amino-acids peptides. With the cleavage efficiency being influenced by the flanking regions [35,36], special attention must be paid to the epitope directly neighboring. It is known that small amino-acid residues such as glycine or alanine favor the antigen processing by the proteasome [37]. Here, we genetically inserted the MHC-I A2L epitope flanked on both sides by “GSGG” linkers in the “VL” exposed loop for ADD A2L or in the “RGD” exposed loop for ADD KGE/RGD +/− RBD.

After demonstrating that ADDomers could be internalized differentially by dendritic cells depending on their design as well as on the DC subset (Figure 2), we studied the impact of ADD Ø and ADD A2L on human DCs subsets in order to determine whether ADDomers could activate them (Figure 3). It was observed that both ADDomers were not toxic for DCs from healthy donors. Both of them also trigger a high activation of BDCA1, BDCA2 and BDCA3 subsets, highlighted by the up-regulation of the three costimulatory molecules (CD40, CD80, CD86), revealing the potent adjuvant properties of the ADDomer itself. Once activated, DCs express costimulatory molecules at their surface which are required for the activation of T cells. CD40 interacts with CD40 ligand on CD4 T cells surface, and CD80/CD86 interact with CD28 on CD8 T cells. Since the ADDomer derives from adenovirus, it could bind to existing receptors as well as trigger receptor cross-linking, which could explain the very high activation of DCs. Regarding cytokine expression, ADDomers (mostly ADD A2L) triggered a slight production of DC-specific cytokines, especially IFNα, IL28, IL29, which are crucial for subsequent antitumor effector elicitation.

Next, we explored the induction of tumor-antigen specific immune response in vitro in human with ADD A2L in other words, we investigated the ability of ADDomers to trigger anti-MelA_26–35_ CD8+ T cells from PBMCs, over 20 days (3 stimulations—one per week) by measuring the percentage of anti-MelA_26–35_ CD8+ T cells every 7 days using dextramer labeling.

This result highlighted the ability of ADD A2L to trigger anti-MelA_26–35_ CD8+ T cells amplification. Moreover, the amplified anti-MelA_26–35_ CD8+ T cells showed a high affinity for the HLA/MelA_26–35_ complex (Figure 5), highlighting their ability to kill target cells with low levels of peptide/MHC complexes.

It was shown in previous work that the genetic insertion of short epitopes in the two exposed loops of ADDomer did not affect the overall particle structure [12]. However, an epitope-based vaccine presents a major limitation for human use: HLA restriction. Indeed, the encoded epitopes are specific to a certain HLA allele. In our case, the epitope A2L is specific to the HLA A2, which is present in only 40% of the Caucasian population. To overcome this limitation, developing a full antigen-based vaccine enabling epitope presentation in all individuals is preferable (i.e., developing an off-the-shelf vaccine) [38]. Unfortunately, genetic insertion of large-folded antigens results in subunit misfolding and insolubility [39]. In a previous study, Villegas et al. have shown that OVA_248–376_ antigen could be fused to WW-domains, allowing interactions with the adenovirus PPxY sequences included in the adenovirus penton base [16,40]. However, this non-covalent interaction relies on a relatively low K_D_ (65 nM) [41] that could not warrant a homogenous batch required for clinical trials. To overcome this limitation, insertion of a SpyTag (13 residues) in the exposed loops of ADDomer has been reported to permit the spontaneous and covalent attachment of large and correctly folded antigens such as the SARS-CoV-2 Receptor Binding Domain or ovalbumine to the particle [15,42]. Fusion of melanoma tumor antigen (MelA) to the SpyCatcher successfully resulted in the display of this antigen to the vaccine platform (Figure 1A,B).

Comparison of ADD MelA versus ADD A2L internalization by DCs subsets was then verified. Indeed, one known mechanism of ADDomers inside cells relies on the binding of integrins with the RGD motif exposed on ADDomer’s “RGD” loops [22,23]. These “RGD” motifs could be shielded by the addition of MelA-SC on the ADDomer’s surface. The results showed that ADD MelA is less but still internalized by all DC subsets compared to ADD A2L (Figure 2). Interestingly, whereas cDC2s strongly fix/capture both ADDomers, cDC1s were a little less efficient, and pDCs even less.

Cross presentation comparison between ADD MelA and ADD A2L was also performed, revealing that ADD MelA seems to be more cross-presented than ADD A2L (Figure 4). This suggests that displaying a protein antigen containing multiple MHC-I and MHC-II epitopes instead of a single epitope only favors the cross presentation of this epitope. An explication to this finding could be the activation of the CD4 immune response by the full-length antigen MelanA, which potentializes the CD8 response against the A2L epitope.

The second part of this study was to specifically address the ADDomers to the dendritic cells via ADDomer’s design modifications. The aim is to limit off-target effects and to enhance cross priming via enhanced ADDomer uptake by DCs.

The de-targeting was performed by mutating the specific integrin binding motif “RGD” motif in the RGD loop into “KGE”, therefore limiting an RGD-dependent-integrin-mediated ADDomer uptake by epithelial cells, which has been observed in previous studies [22].

The re-targeting was achieved by displaying on the ADDomer’s surface the Spike Receptor Binding Domain of SARS-CoV-2 fused to the SpyCatcher (RBD-SC). This was carried out via the SpyTag/SpyCatcher system described earlier [15]. RBD-SC is secreted by insect cells during its production in baculovirus expression system and is therefore glycosylated, mostly by poly-mannoses [24,25,26], which can be recognized by C-type lectin receptors (CLRs) such as DC-SIGN and MGL present on the dendritic cell surface [29,30]. The A2L epitope was then moved from the VL to the RGD loop next to the RGD/KGE motif (Figure 1A).

To evaluate first de-targeting efficacy, A549 adherent cells were incubated with ADD KGE and ADD RGD for 1h. Internalization was evaluated by flow cytometry and microscopy. Both techniques show that ADD KGE is less but still internalized by A549 cells than ADD RGD suggesting an efficient de-targeting (Figure 6). Moreover, the microscope experiment suggests that ADD RGD and ADD KGE might use different internalization mechanisms. ADD KGE seems therefore to be internalized by a non-RGD-dependent-integrin-mediated mechanism.

Secondly, the re-targeting via the interaction of the RBD-SC with CLRs was evaluated by SPR. RBD binding to captured DC-SIGN and MGL CLRs via the glycosylation patterns was measured. This experiment revealed that compared to ADD KGE RBD and RBD-SC, ADDomer not harboring RBD (ADD KGE) has very poor interaction with the surfaces. On the contrary, the compounds containing RBD interact strongly with the surfaces, thanks to strong affinities to CLRs. We also observed a logical higher affinity for ADD KGE RBD due to the more important avidity brought by the multivalent presentation of RBD by the ADDomer platform (Figure 7). The affinity of DC-SIGN for these compounds is logically stronger than for MGL, since glycosylations are principally composed of poly-mannoses (present on the RBD N-glycosites when produced in insect cells) [24,25,26], which are the natural ligands of DC-SIGN [29]. MGL, however, has a better affinity for N-acetyl galactosamine (putatively present on the RBD O-glycosites when produced in insect cells) [26,30].

When we looked at the re-targeting at a cellular level, we observed that the addition of RBD did not enhance ADDomer’s uptake as expected and on the contrary, reduced the proportion of ADDomers internalized by the dendritic cells (Figure 8). For ADD RGD versus ADD RGD RBD, just as for ADD MelA, this decrease in internalization can be caused by the shielding of ADDomer’s integrin binding motif (RGD motif in the RGD loop) by the RBD-SC on the ADDomer’s surface. For ADD KGE versus ADD KGE RBD, as mentioned for A549 cells, internalization seems to take place via a non-RGD-dependent-integrin-mediated mechanism. One can imagine that this mechanism involves recognition of other molecular patterns present on the ADDomer’s surface which are also shielded by the RBD-SC. However, in both cases (KGE versus RGD), the internalization seems to be mediated by an unknown receptor (putatively a CLR) present on the dendritic cell surfaces (Figure 7). In terms of cross-presentation, our results showed that ADD RGD is cross-presented by dendritic cells contrary to ADD KGE, ADD KGE RBD and ADD RGD RBD. This suggests that the RGD-dependent-integrin mediated internalization might play a role in the cross-presentation process of the ADDomer’ displayed epitope A2L. This is also consistent with the observation that the ADDomer RGD is fixed in a higher level by cDC1s compared to ADDomer KGE and RBD, and cDC1s are the subset of DCs exhibiting the highest cross-presentation capacities.

Altogether, our results show that ADDomer could be a promising cancer vaccine platform displaying tumor antigens and epitopes. Epitopes can be genetically inserted into the ADDomers loops without degrading the ADDomer structure. On the other hand, antigen insertion requires the expression of both ADDomer SpyTag and the antigen fused to the SpyCatcher. This covalent and autocatalytic binding allows the production of a “ready to use” cancer vaccine applicable to a large population and to different types of cancers with known epitopes and antigens. Currently, cancer vaccines use either tumor-associated antigens or more personalized neo-antigens which could both be displayed by the ADDomer platform. Moreover, the multiple epitopes and antigens could be displayed at the same time on ADDomers, allowing a multi-specific immune response and therefore limiting treatment failure or resistance.

## Data Availability

Data are available upon request.

## Supplementary Materials

The following supporting information can be downloaded at: https://www.mdpi.com/article/10.3390/biomedicines10112881/s1, Figure S1: Separation of the complex ADD-mCherry from the left over mCherry-SC. (A) SDS PAGE gel of boiled and reduced samples showing left over mCherry-SC after incubation of ADD ST with mCherry-SC. (B) SDS PAGE gel of boiled and reduced samples and sucrose gradient image showing the separation of the complex ADD mCherry and the left over mCherry-SC; Figure S2: Preparation of ADDomers labeled with Alexa 647 for ADDomers internalization evaluation by DC subsets. (A) Workflow of ADDomers AF647 preparation and purification. (B) Agarose gel of labeled and non-labeled ADD mCherry; Figure S3. Dotplots of ADDomer fixation on DC subsets. (A) Gating strategy of DC subsets. (B) ADDomer fixation on DC subsets; Figure S4: ADD Ø and ADD A2L cross priming evaluation. (A) dextramer. (B) Workflow of ADD Ø and ADD A2L cross priming evaluation. (C) Dot blots showing the percentage of CD8+ Dext MelA+ T cells at D0 and at D20 for each condition (dot plots pre-gated on CD45+ CD3+ CD8+ T cells); Figure S5: Zoom on ADD KGE RBD binding evaluation on DC-SIGN by surface plasmon resonance. Separate fitting of ADD KGE RBD with DC-SIGN.
