# Supplementary material for: Adenovirus-Inspired Virus-like-Particles Displaying Melanoma Tumor Antigen Specifically Target Human DC Subsets and Trigger Antigen-Specific Immune Responses
Source: Biomedicines. 2022 Nov 10;10(11):2881. doi: 10.3390/biomedicines10112881 (PMC9687312; doi:10.3390/biomedicines10112881)
Supplement: Supplementary file 1 [file biomedicines-10-02881-s001.zip › biomedicines-1941123-supplementary.pdf]

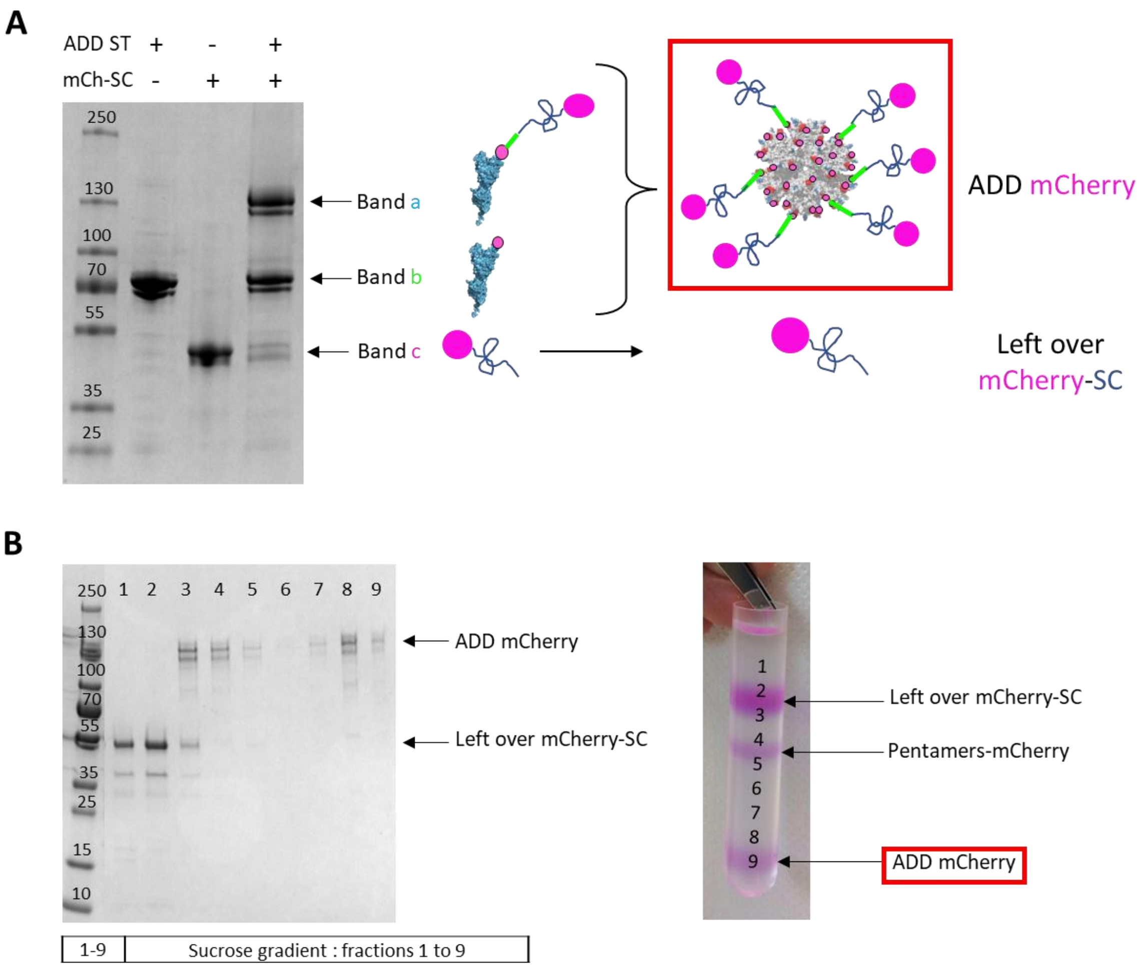

Supplementary figure S1: Separation of the complex ADD-mCherry from the left over mCherry-SC

(A) SDS PAGE gel of boiled and reduced samples showing left over mCherry-SC after incubation of ADD ST with mCherry-SC (B) SDS PAGE gel of boiled and reduced samples and sucrose gradient image showing the separation of the complex ADD mCherry and the left over mCherry-SC

A

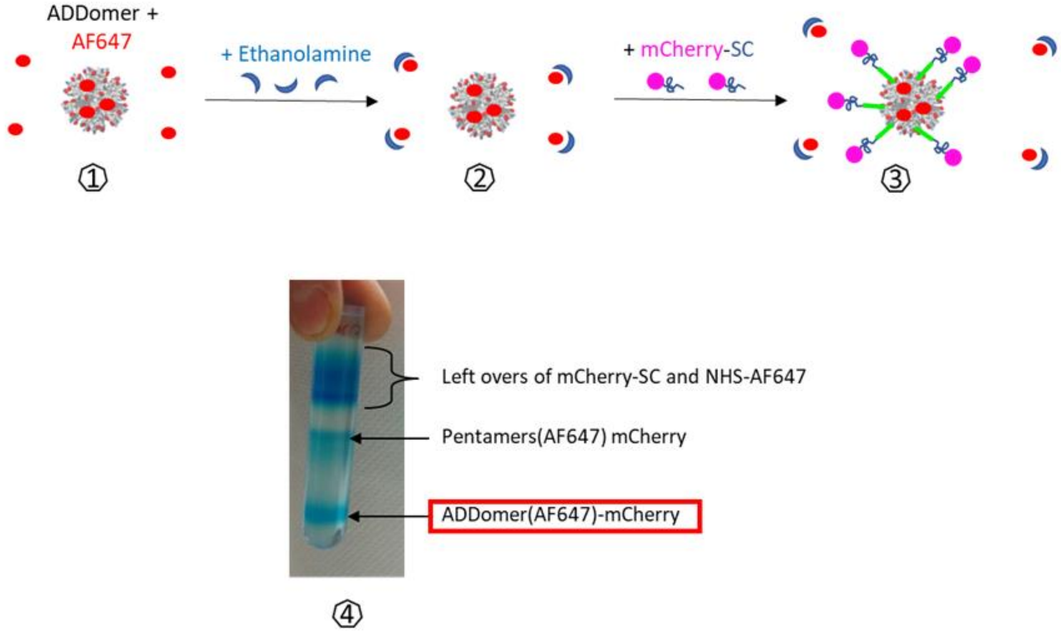

B

1	2	3	4	5
mCherry-SC<br>Crt +	RBD-SC<br>Crt -	MelanA-SC<br>Crt -	ADD ST (AF647)	ADD ST (AF647)<br>mCherry

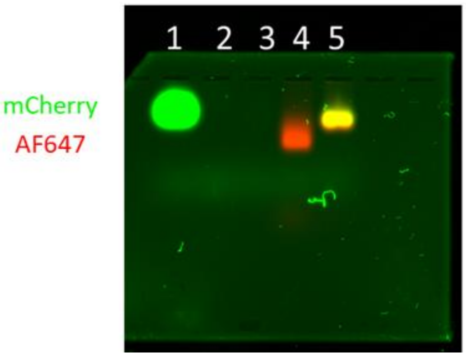

Supplementary figure S2: Preparation of ADDomers labelled with Alexa 647 for ADDomers internalisation evaluation by DC subsets

(A) Workflow of ADDomers AF647 preparation and purification (B) Agarose gel of labelled and non-labelled ADD mCherry.

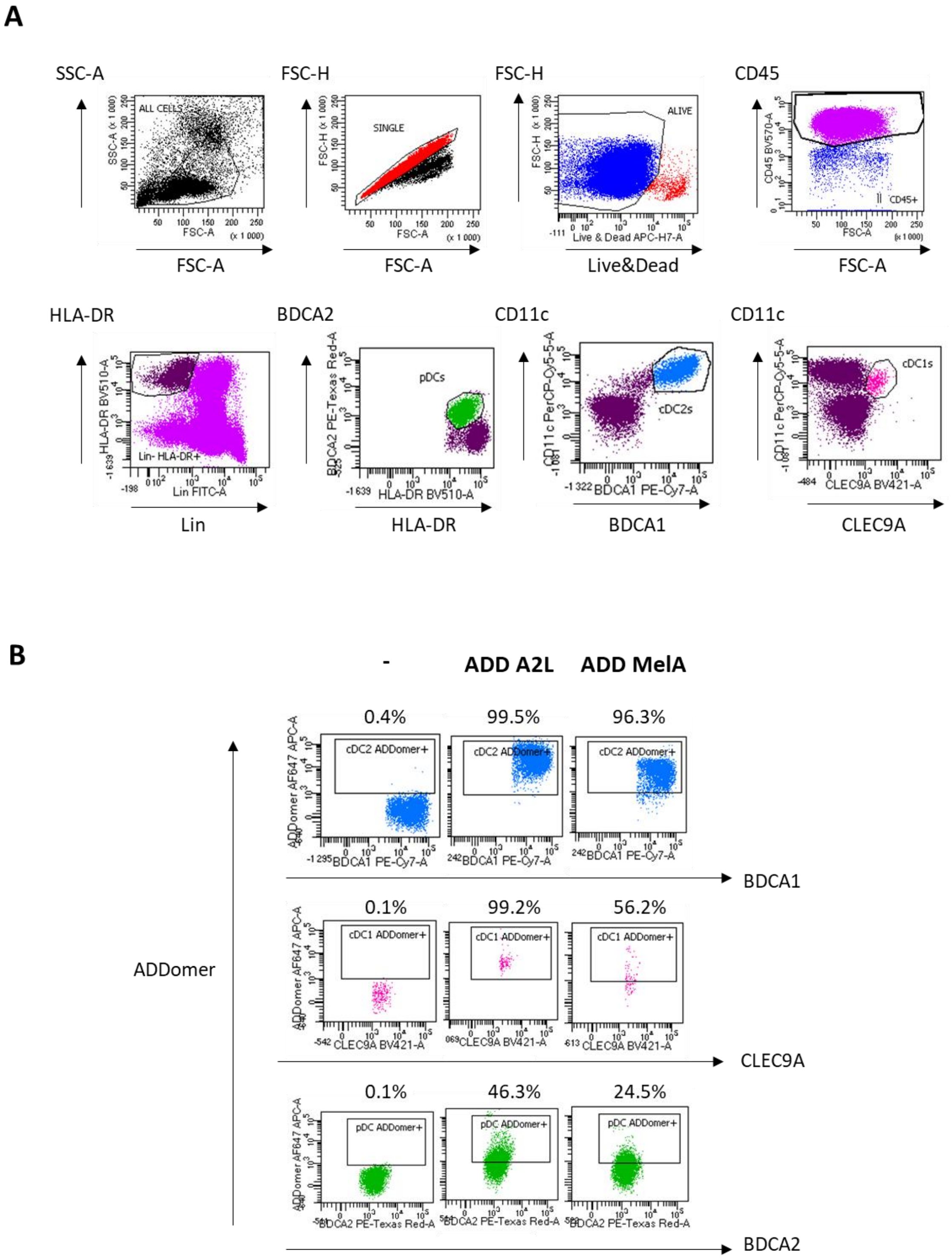

Supplementary figure S3: Dotplots of ADDomer fixation on DC subsets

(A)Gating strategy of DC subsets (B) ADDomer fixation on DC subsets.

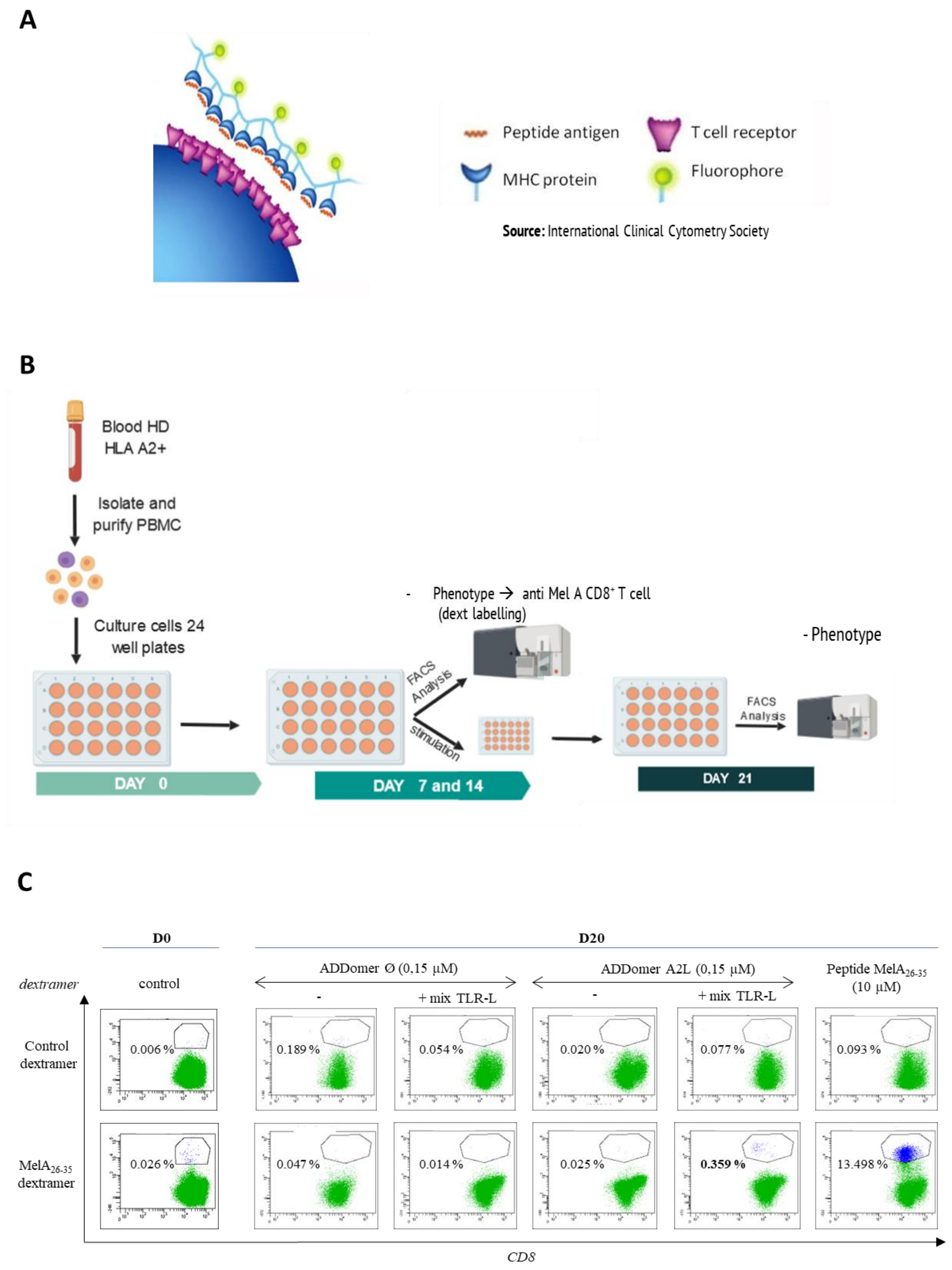

Supplementary figure S4: ADD Ø and ADD A2L cross priming evaluation

(A) dextramer (B) Workflow of ADD Ø and ADD A2L cross priming evaluation (C) Dot blots showing the percentage of CD8+ Dext MelA+ T cells at D0 and at D20 for each condition (dot plots pre-gated on CD45+ CD3+ CD8+ T cells).

ADD KGE RBD interaction with **DC-SIGN**

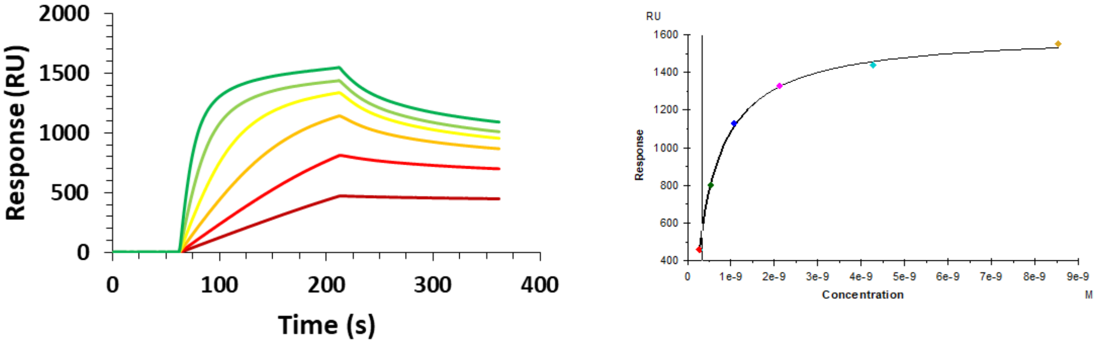

Steady State affinity

$K_{D\ app}$ 0.34 nM +/- 0.046 nM

Supplementary figure S5: Zoom on ADD KGE RBD binding evaluation on DC-SIGN by surface plasmon resonance

Separate fitting of ADD KGE RBD with DC-SIGN.
